# Measuring and modelling microclimatic air temperature in a historically degraded tropical forest

**DOI:** 10.1007/s00484-022-02276-4

**Published:** 2022-03-31

**Authors:** Christopher D. Marsh, Ross A. Hill, Matthew G. Nowak, Emma Hankinson, Abdullah Abdullah, Phillipa Gillingham, Amanda H. Korstjens

**Affiliations:** 1 Department of Life and Environmental Sciences, Bournemouth University, Poole, UK; 2 Department of Biology, University of New Mexico, Albuquerque, NM USA; 3 The PanEco Foundation – Sumatran Orangutan Conservation Programme, Chileweg 5, Berg Am Irchel, Switzerland; 4 Department of Anthropology, Southern Illinois University, Carbondale, IL USA; 5 Department of Biology, Universitas Syiah Kuala, Banda Aceh, Aceh, Indonesia

**Keywords:** Microclimate, UAV, Canopy structure, Rainforest, Remote sensing

## Abstract

Climate change is predicted to cause widespread disruptions to global biodiversity. Most climate models are at the macroscale, operating at a ~ 1 km resolution and predicting future temperatures at 1.5–2 m above ground level, making them unable to predict microclimates at the scale that many organisms experience temperature. We studied the effects of forest structure and vertical position on microclimatic air temperature within forest canopy in a historically degraded tropical forest in Sikundur, Northern Sumatra, Indonesia. We collected temperature measurements in fifteen plots over 20 months, alongside vegetation structure data from the same fifteen 25 × 25 m plots. We also performed airborne surveys using an unmanned aerial vehicle (UAV) to record canopy structure remotely, both over the plot locations and a wider area. We hypothesised that old-growth forest structure would moderate microclimatic air temperature. Our data showed that Sikundur is a thermally dynamic environment, with simultaneously recorded temperatures at different locations within the canopy varying by up to ~ 15 °C. Our models (*R*^2^ = 0.90 to 0.95) showed that temperature differences between data loggers at different sites were largely determined by variation in recording height and the amount of solar radiation reaching the topmost part of the canopy, although strong interactions between these abiotic factors and canopy structure shaped microclimate air temperature variation. The impacts of forest degradation have smaller relative influence on models of microclimatic air temperature than abiotic factors, but the loss of canopy density increases temperature. This may render areas of degraded tropical forests unsuitable for some forest-dwelling species with the advent of future climate change.

**Supplementary Information:**

The online version contains supplementary material available at 10.1007/s00484-022-02276-4.

## Introduction

Habitat modification and climate change are among the primary threats to global biodiversity (Nowakowski et al. 2018). Land surface temperatures in tropical rainforest regions have risen by ~ 0.25 °C per decade since the mid-1970s (Malhi and Wright 2004) and are projected to rise by 3–8 °C over the twenty first century (Malhi et al. 2009). Forest degradation, measured by partial canopy cover loss, affected 185 million ha between 2000 and 2012, with over 156 million ha of that occurring within tropical forests (van Lierop et al. 2015). Habitat modification affects landscape-scale variations in climate, which leads to the synergistic effects of these two drivers (Todd and Andrews 2008; Arroyo-Rodrigues et al. 2016; Tuff et al. 2016). Whilst organisms are experiencing both habitat modification and climate change simultaneously, these threats are typically studied independently (Sirami et al. 2017, Oliver and Morecroft 2014, but see Senior et al. 2017).

Ecologists aiming to understand and predict the influence of climate on species often use models based on macroclimatic variables, which are generally interpolated from data measured by standard meteorological stations at 1.5–2 m above ground (Fick and Hijmans 2017). However, organisms experience climate at a micro-scale, and temperature variation is highly scale-dependent (Chen et al. 1999). Due to the influence of vegetation structure, microtopography and soil, microclimate experienced at the local level may only be weakly correlated with macroclimate (Graae et al. 2012; WallisDeVries et al. 2011; Potter and Hargrove 2013). Mobile organisms can move within landscapes which incorporate a broad range of microclimates, allowing them to maintain their optimum climatic conditions (Oliver and Morecroft 2014) across tens or hundreds of metres per day, depending on body size and mobility (Jenkins et al. 2007). In forested landscapes, canopy structure, rocks and logs provide a spatial and temporal mosaic of temperatures, which terrestrial species may be able to exploit for thermoregulation and to buffer against more extreme temperatures (Scheffers et al. 2014a, b). For arboreal species, which make up ~ 76% of vertebrate species in tropical forests (Kays and Allison 2001), movement is in three dimensions, so forest canopy structure and topographical variation can be utilised to buffer against solar radiation.

Microhabitats have been shown to buffer temperatures in a consistent manner within forest systems and can reduce extreme heat exposure by up to 10 °C (Scheffers et al. 2014b). In a degraded forest, the availability of microhabitats and the effectiveness of their thermal buffering are influenced by varying levels of vegetation density (Pringle et al. 2003). The level of degradation, time since degradation and plant species establishment will affect forest structure and therefore microclimate (Pohlman et al. 2007; Norris et al. 2012; Harper et al. 2005; Brokaw 1982; Mulkey and Pearcy 1992; Laurance et al. 2006). In Colombia, thermally buffered microhabitats were shown to increase in abundance with forest succession, from young secondary forest to primary forest (González del Pliego et al. 2016).

Forest degradation creates a dynamic, three-dimensional thermal environment which varies across daily and seasonal cycles. In a degraded forest, changes in vegetation structure due to selective logging may alter the microclimate environment across relatively small distances, both vertically and horizontally, which recently produced microclimate modelling frameworks are unable to account for as they lack inputs for complex vegetation (Kearney and Porter 2017). Thermal variation is not restricted to horizontal variation in vegetation composition but also occurs vertically among canopy strata (Scheffers et al. 2017). Therefore, to measure within-canopy microclimate accurately across a degraded forest landscape, at the small scales that most species experience, requires new and innovative methods. Despite the long history of microclimatology (Potzger 1939), it is only more recently that developments in technology and advances in computing power have made it possible to take simultaneous measurements over large areas (Jones 2013; Wang et al. 2013). However, measuring small-scale microclimate fluctuations across a large area in structurally complex degraded forest habitats still presents significant challenges. Detailed three-dimensional vegetation surveys over large areas have previously been prohibitively expensive for most researchers (Hummel et al. 2011), with light detection and ranging (LiDAR) from small aircraft and time-consuming terrestrial surveys being the only options to obtain these data accurately (Hill and Hinsley 2015). With the advent of unmanned aerial vehicles (UAVs) used in an ecological context (Koh and Wich 2012; Anderson and Gaston 2013; Zahawi et al., 2015) and structure-from-motion photogrammetry software, measurements of canopy topography are now accessible and cost-effective (Alexander et al., 2017, Lisein et al. 2013, Wich et al. 2015, Zellweger et al. 2019). Simultaneously, data logging hardware (i.e. measurement systems which independently record data points at set times) are now available at relatively low cost, allowing microclimate data to be recorded independently across a network (Hubbart et al. 2005). Combining these technologies enables the development and testing of a three-dimensional microclimate model that incorporates vegetation structure across a wide landscape (Eckmann et al. 2018; Zellweger et al. 2019).

This study explores the effects of vegetation structure on microclimatic air temperature within a degraded tropical forest to enable landscape-wide prediction within forest canopies across hourly time periods. We carried out detailed and wide-ranging vegetation surveys (both field- and UAV-based) combined with in situ air temperature measurements at the microhabitat scale. We hypothesise that degraded areas of forest, identifiable by low values in variables associated with old-growth forest (such as trunk basal area and crown area), will show increased temperatures at all canopy heights across hourly time periods. Additionally, we expect that degraded areas of forest will be identifiable by UAV surveys, allowing the construction of a model that allows predictions of temperature at the scale of tens of metres. This will enable a detailed understanding of how vegetation structure, as measured by UAV or ground measurements, affects microclimate air temperature profiles.

## Materials and methods

### Study site

**Fig. 1:**
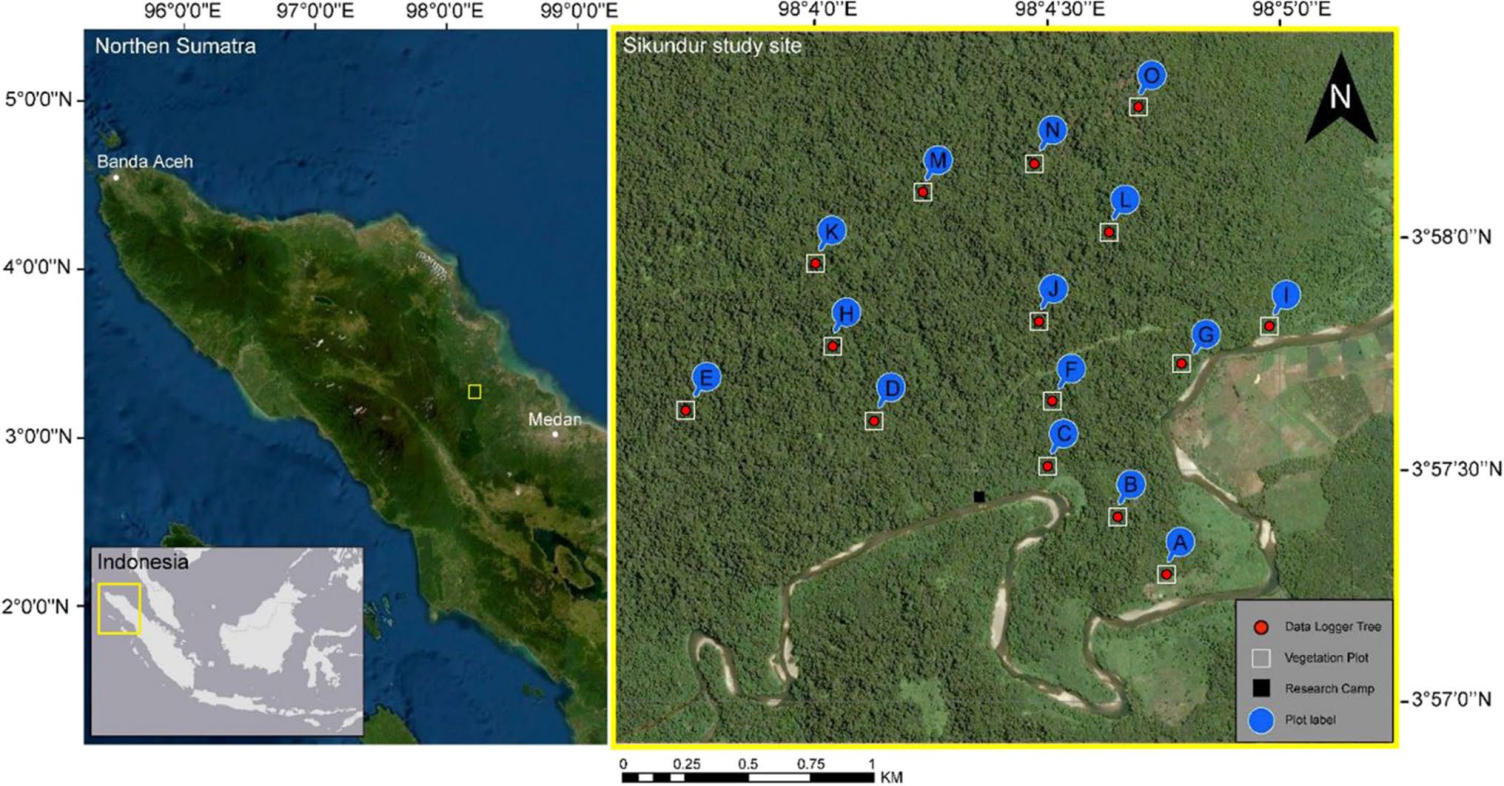
Data logger and vegetation plot locations within Sikundur in Northern Sumatra, Indonesia

The Sikundur study site (before 1980 known as the Sikundur Reserve, est. 1938) covers ca. 3 × 3 km of the Leuser Ecosystem (latitude, 3.95 N; longitude, 98.07 W) in Northern Sumatra, Indonesia (Fig. 1). This site has been the focus of previous research projects on primates and emergent trees (Knop et al. 2004, Askew and Morrogh-Bernard 2016; Alexander et al. 2018 Harrison et al. 2020, Hankinson et al. 2021), but none has previously considered microclimate, which remains relatively under-studied in tropical environments. Sikundur is one of the last remaining sections of lowland rain forest in Sumatra. The Leuser Ecosystem is the only remaining area where Sumatran orangutans (*Pongo abelii*), elephants (*Elephas maximus sumatranus*), tigers (*Panthera tigris sumatrae*), rhinos (*Dicerorhinus sumatrensis*) and sun bears (*Helarctos malayanus*) still co-exist (Hitchcock and Meyers 2006) and are a significant part of the UNESCO listed ‘Tropical Rainforest Heritage of Sumatra’. It has also been declared a National Strategic Area due to the ecosystem services that it provides.

The Sikundur site was selectively logged from the late 1960s, which continued and progressively intensified in some areas until the 1980s. During the logging operations from the late 1960s until 1982, an average of 11 large trees per hectare was felled (Knop et al. 2004). Five years after the end of the logging operation, Abdulhadi et al. (1987) found that 54% of the remaining trees showed some damage caused by the logging. Following the establishment of the Gunung Leuser National Park in 1980, logging in the Sikundur area continued primarily at the park border. The average monthly temperature in Sikundur (as recorded by weather station) is 27.3 °C, ranging from 26.1 °C in November to 29.2 °C in April (Nowak, 2015). Average monthly rainfall is 256.4 mm, ranging from 12.4 mm in February to 535.4 mm in November (Nowak, 2015). As Sikundur is within 4° of the equator, sunrise and sunset occur at 6:25 am and 6:25 pm (± 17 min) throughout the year, and there is little thermal seasonality.

### Vegetation plots

We surveyed fifteen 25 × 25 m fixed plots in March 2016 (Fig. 1). Plot centre locations were randomly generated using ArcMap (version 10.4) located at least 500 m apart (Fig. 1), with plot corners located in the field using a handheld GPS unit with a positional accuracy of ~ 5 m (Garmin GPSMAP 64 s). Within each plot, for every tree larger than 10 cm diameter at breast height (DBH, Ganzhorn et al 2011), the following variables were recorded: DBH, tree and bole (1st major branch) height (measured using a ‘Nikon Forestry Pro’ laser range finder/hypsometer, see https://imaging.nikon.com/lineup/sportoptics/laser/forestrypro/https://www.forestry-suppliers.com/Documents/1345_msds.pdf), crown width (measured with a tape measure as the distance, in metres, at the cardinal compass points from the trunk to the edge of the crown), tree crown connectivity with surrounding trees (estimated as a percentage) and the number of branches within five size categories (< 2 cm, 2–4 cm, 4–10 cm, 10–20 cm, > 20 cm in diameter) (Manduell et al. 2012) (Supplementary Materials, Fig. 1). The following plot-level variables were derived: Lorey’s height (mean tree height weighted by basal area), mean tree height, tree height range, mean bole height, plot (or total) basal area, mean DBH, quadratic mean diameter (the square root of the arithmetic mean of squared values), number of stems, mean and cumulative crown area, mean crown connectivity, mean branch counts of each category and a canopy density estimation using a mean of four photographic measurements at the plot corners analysed using CanopyDigi software (Goodenough and Goodenough 2012).

### Microclimate data loggers

To record fluctuations in temperature, microclimate data loggers (HOBO UA-002–08 8 K Pendant Temperature/Light Logger, with an accuracy of ± 0.47 °C) were placed below the canopy within vegetative shade at various heights within 15 trees, each within the fixed plots (Supplementary Materials, Fig. 1). The microclimate data loggers recorded temperatures at 60-min intervals between March 22nd, 2016, and November 29th, 2017. Sunshades were not used with the data loggers as these have been shown to alter air temperature measurements (Richardson et al. 1999; Bramer et al. 2018), resulting in the recording of shade temperature as opposed to the microclimate which forest organisms would be exposed to (Brock et al. 1995). To counter the potential effects of direct solar radiation causing bias on air temperature recordings a simple algorithm was used to flag likely errors (following Frey et al. 2016). Data logger recordings of ≥ 32,000 lx (the level of direct sunlight) (Hiscocks 2011) were flagged using R (R Core Team 2019). The full 24-h period in which a flagged data logger recording occurred was removed from the data set (i.e. if a reading of ≥ 32,000 lx was detected at 3 pm on day 89 of logger deployment, recordings from 1 to 12 am of day 89 were deleted). This resulted in 3223 data points from 134 logger days (i.e. 1.09% of total recordings) being removed before analysis.

One to three data loggers were placed in a single tree at the approximate centre of each vegetation plot (two data loggers if the tree was up to 20 m in height, and three if the tree was ≥ 20 m; however, during the course of the study, three trees (labelled as B, J and O) only had one logger for some of the time due to logger failures). The first logger was placed as high as possible using a modified ‘big shot’ catapult and rope system, and the other data logger(s) were placed below it at approximately 10 m vertical increments. This vertical distance between loggers was initially measured in the rope length between loggers, although due to tree branches intersecting the rope, when data logger heights were confirmed using a ‘Nikon Forestry Pro’ laser range finder/hypsometer, vertical distances between loggers were found to range from 6 to 10 m. Between April and August 2016, 30 loggers were recording simultaneously in the 15 trees. From August 2016 to June 2017, 19 data loggers had to be removed due to budgetary restrictions, leaving 11 remaining loggers recording in 6 trees (see Supplementary Materials, Fig. 9). In June 2017, 18 data loggers were placed in 8 of the original 15 trees, with the same vertical distances between loggers, although identical heights from the ground were difficult to replicate. For this reason, all individual data logger recordings (*n* = 52) were treated as separate recordings for analysis, as their heights, aspect and shading could not be replicated exactly. Overall, the highest microclimate data logger was 35.5 m above ground level, with the mean height of loggers being 16.6 m. In addition to daytime air temperature, rainfall was manually measured daily at the research station using a rain gauge.

For the purpose of analysis, daytime air temperature recordings of each data logger were summarised by hour (8 am to 6 pm) by month as both the mean and the 95th percentile of recordings (as maximum temperature recordings are often outliers and possible artefacts of measurement error) (Maclean et al., 2021) for a total of 3853 data points.

### Unmanned aerial vehicle surveys

To record aerial imagery of the study site, we used a fixed wing UAV. The modified ‘Skywalker’ UAV (see Supplementary Materials, Fig. 2) was controlled manually (i.e. radio-controlled) for take-off and landings and switched to autopilot to fly along pre-set ‘lawnmower’ routes (see Supplementary Materials, Fig. 3) programmed using Mission Planner software (version 1.3.58). A digital camera (SONY RX100 mk4) took RGB photographs at GPS locations controlled by the autopilot and a Seagull #MAP 2 switch, ensuring 80% overlap of each photograph, both in the direction of UAV travel and between flight paths (i.e. ‘sidelap’). Eleven flights over the Sikundur area were flown between June 13th and 16th, 2017, at an altitude of ~ 200 m above ground level, covering a total area of ~ 26 km^2^ with a ground sampling distance of 4.82 cm per pixel. A total of 4811 geotagged images taken during these flights were processed in Agisoft Photoscan software (version 1.2.0.2152, now *Agisoft Metashape*) to create a digital surface model (DSM) of the forest canopy, with a spatial resolution of 23.1 cm^2^ per cell. The DSM was 24,525 × 25,576 cells in size and provided the elevation (in metres above sea level) for the topmost surface per cell (Cunliffe et al., 2016) (see Supplementary Materials, Fig. 4).

**Fig. 2:**
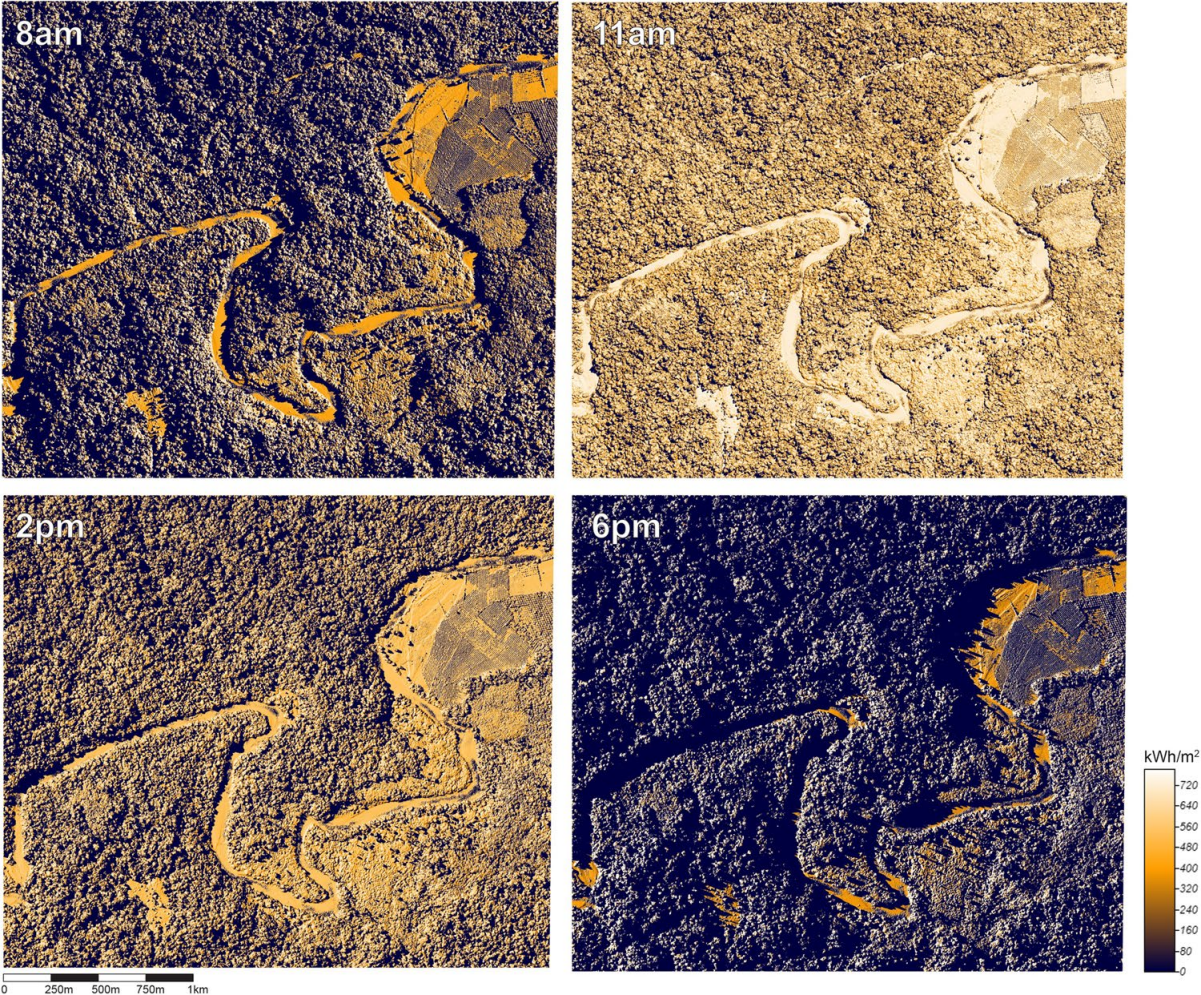
Examples of hourly estimates of potential incoming solar radiation on a section of the Sikundur site. Note the shadowing effects of terrain and vegetation

**Fig. 3:**
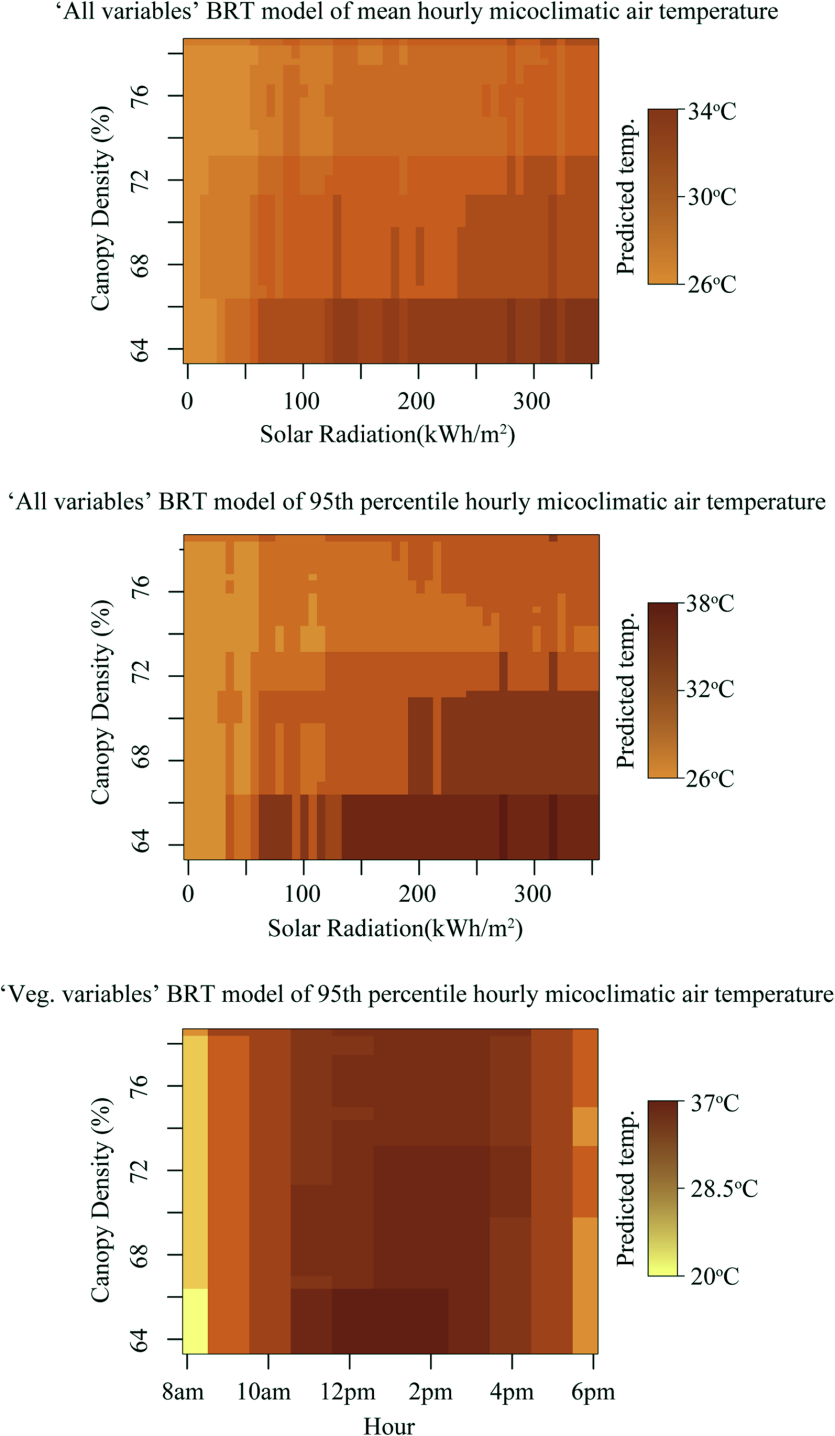
Predicted temperature as a product of variable interactions between canopy density, solar radiation and hour in three boosted regression tree models depicted with other variables in models set to their mean values

### Variables derived from UAV surveys

Potential incoming solar radiation, measured in kWh/m^2^, was estimated across the study area using the UAV-derived DSM of the forest canopy and the Potential Incoming Solar Radiation tool in System for Automated Geoscientific Analyses (SAGA) software (version 2.3.2; Conrad et al. 2015). The resulting layers provide a geospatial–temporal raster estimating how much solar radiation is reaching the topmost section of each DSM cell based on the position of the sun at each hour, which includes shadow effects of surrounding canopy structure and terrain (Fig. 2). These hourly estimates of potential solar radiation (from 8 am to 6 pm) were calculated for the 16th of each month (summarised to a single day due to computing power demands) for January to December adjusting values as the sun’s zenith changed by month.

To allow direct comparison between data from the UAV-derived DSM and from vegetation plots and to account for inaccuracies in GPS locations both of data logger sites and of UAV imagery used to create the DSM, both the DSM and the derived Potential Incoming Solar Radiation layers were aggregated to a 25 × 25 m grid matched to vegetation plot corner locations as closely as possible before data were extracted for use in microclimatic air temperature modelling. (The maximum discrepancy between grid corners and vegetation plot corners was ~ 5 m.) The DSM was used to produce seven variables of univariate statistics of elevation, namely, ‘minimum’, ‘maximum’, ‘median’, ‘mean’, ‘range’ and ‘standard deviation’, of surface elevation within the aggregated 25 m spatial resolution cells (using the ‘Zonal Statistics’ tool in ArcGIS version 10.4). Additionally, three more variables were derived: (1) ‘upper canopy range of elevations’ (‘maximum’ minus ‘mean’ elevations), (2) ‘lower canopy range of elevations’ (‘mean’ minus ‘minimum’ elevations) and (3) ‘relative height’ (difference between ‘mean’ elevation of the 25 m resolution cell and the mean elevation of a 75 m resolution grid surrounding the 25 m resolution cell). These values were then extracted at the locations of each data logger (using ArcGIS version 10.4), such that data loggers within the same tree had the same vegetation structure data values.

Similarly, the Potential Incoming Solar Radiation was aggregated to a 25 × 25 m grid and extracted, with separate layers created for each hour of the day (8 am through 6 pm) of each month (January to December, for the 16th day of each month) creating a total of 132 layers. For analysis, we used the mean incoming solar radiation value at a given hour across months as variables.

### Statistical analyses

#### Vegetation structure analysis

Vegetation structural differences between plots were examined using a Kruskal–Wallis test. To examine whether field plot and UAV-derived structure variables were correlated, each variable was tested against every other using a Pearson’s correlation as all data were of a Gaussian distribution.

#### The mean and the 95th percentile hourly temperature

To determine the relative contribution of incoming solar radiation and vegetation structure (derived from both plot measurements and UAV surveys) on determining the mean and the 95th percentile hourly microclimatic air temperature recorded by data loggers (*n* = 52), a machine learning approach was used (Frey et al. 2016). Boosted regression trees (BRTs) allow the inclusion of a large number of spatially overlapping variables and potential collinearity between them (Elith et al. 2008). Given that the variables from UAVs were derived from univariate statistics and vegetation plot measurements were often correlated (Dray et al. 2008), this allowed us to explore potential correlates to air temperature without restricting predictor variables (Elith et al. 2008). We used the R programme version 3.6.1 (R Core Team 2017) in combination with the ‘dismo’ package version 0.8–17 (Hijmans et al. 2013) for BRT analyses.

To explore which variables were best at predicting the mean and the 95th percentile hourly air temperature, three models were run, limiting predictor variables to those obtained either from vegetation plots (*n* = 17), UAV surveys (*n* = 13) or from both sources (*n* = 30), with all models including the hour of day and data logger height and mean monthly rainfall as additional variables. We used 70% of recorded microclimatic air temperature recordings to train Gaussian BRT models and then used these model parameters to predict the recorded temperatures of the remaining 30% of data. Initial BRT models were run using the same parameters (interaction depth = 2, learning rate = 0.05, bag fraction = 0.5). These initial models were then simplified, keeping variables with a relative influence of ≥ 0.5% on each model and removing variables with the lowest contributing predictive power (Elith et al. 2008), until models with just ten variables were produced for each ‘mean hourly air temperature’ model. The BRT output produces a final predictive model as well as the relative influence of each variable on the model. This allowed the exploration of which vegetation variables, and at which values, were most influential on mean hourly air temperature.

To assess model accuracy, we used three separate measures. First, the percentage of deviance explained by each mean hourly air temperature model was calculated using the pseudo-determination coefficient *D*^*2*^ (Mateo and Hanselman 2014), using

1 $${D}^{2}=1-\left(residual deviance/total deviance\right)$$

Secondly, *R*^2^ values were produced for each model using best model predictions (defined in tenfold cross-validation) regressed against the ‘hold-out’ data (i.e. 30% of recorded microclimatic air temperature), using simple linear regression. Finally, we calculated the root-mean-square error (RMSE) of predicted and observed hourly air temperature using

2 $$RMSE=\sqrt{\frac{{\sum }_{i=1}^{N}\left({Predicted}_{i}-{Actual}_{i}\right)}{N}}$$

## Results

### Vegetation plots

A total of 380 trees were measured over 15 vegetation plots. DBH ranged from 10 to 116 cm, tree height from 5 to 48 m, bole height from 0.5 to 38 m and crown area from 1.9 to 236.5m^2^ (Supplementary Materials, Figs. 6 and 7). Variables associated with old-growth forest (high values in DBH, tree height, crown area) (Frey et al. 2016) were positively correlated (DBH ~ tree height, *r* = 0.73, *p* < 0.005, DBH ~ crown area, *r* = 0.75, *p* < 0.005, tree height ~ crown area, *r* = 0.62, *p* < 0.005) (Supplementary Materials, Fig. 5). Nearly all structural variables were significantly different between vegetation plots (see Table 1), with the exception of DBH measurements, branch count diameter > 20 cm and plot basal area, making it problematic to define areas of forest as ‘intact’ or ‘degraded’ (see Supplementary Materials, Figs. 6 and 7).

**Table 1:** Results of Kruskal–Wallis one-way analysis of variance test of vegetation variables across the 15 vegetation plots, with variables that differ between plots marked with * for *p* < 0.05 or ** for *p* < 0.001

Variable	*X*^2^	d.f	*p*
Tree height	47.52	14	< 0.001**
Bole height	65.33	14	< 0.001**
Branch count diam. > 20 cm	20.99	14	0.101
Branch count diam. 10–20 cm	26.93	14	0.02*
Branch count diam. 4–10 cm	24.51	14	0.04*
Branch count diam. 2–4 cm	59.4	14	< 0.001**
Branch count diam. < 2 cm	73.43	14	< 0.001**
Connectivity	81.15	14	< 0.001**
Crown area	27.89	14	0.014*
DBH	18.22	14	0.197
Plot basal area	18.38	14	0.19

Some sites had signs of historic logging (i.e. tree stumps, cut sections of tree), but leaf litter and decomposition made the assessment of their abundance within a given area unreliable.

### Microclimatic air temperature recordings

Temperatures recorded by data loggers ranged from 19.47 to 46.21 °C after data were cleaned, with the mean temperature across the entire time period across all loggers being 26.52 ± 3.51 °C, a mean daytime (from 8 am to 6 pm) temperature of 28.54 ± 3.47 °C and a mean night-time temperature of 24.51 ± 1.31 °C (see Supplementary Materials, Fig. 8).

We recorded a wide range of temperatures simultaneously, with recordings at the same hour of the day varying by as much as 15.2 °C between data loggers across the study site and by as much as 14.8 °C between data loggers at different heights in the same tree at the same time. Across all data loggers, the hottest part of the day on average was 2 pm, although temperature peaks at different locations ranged from 9 am to 5 pm (see Supplementary Materials, Fig. 10a–d).

### Relationships between UAV-derived DSM variables and ground plot variables

UAV-derived variables at the site of vegetation plots, either extracted directly from the aggregated DSM or modelled from it (i.e. potential incoming solar radiation), were moderately correlated with ground field plot variables associated with old-growth forests (Table 2) (Frey et al. 2016). For example, ‘range of elevations’ and ‘lower canopy range’ were significantly correlated with six plot variables: ‘mean tree height’ and ‘range of elevations’ (*r* = 0.59, *p* = 0.019), ‘mean bole height’ and ‘range of elevations’ (*r* = 0.52, *p* = 0.048), ‘mean connectivity’ and ‘range of elevations’ (*r* = 0.53, *p* = 0.041), ‘quadratic mean diameter’ and ‘lower canopy range’ (*r* = 0.56, *p* = 0.028), ‘mean branches > 20 cm diameter and ‘lower canopy range’ (*r* = 0.53, *p* = 0.041) and ‘mean branches 20–10 cm diameter and ‘lower canopy range’ (*r* = 0.60, *p* = 0.018) (Table 2).

**Table 2:**
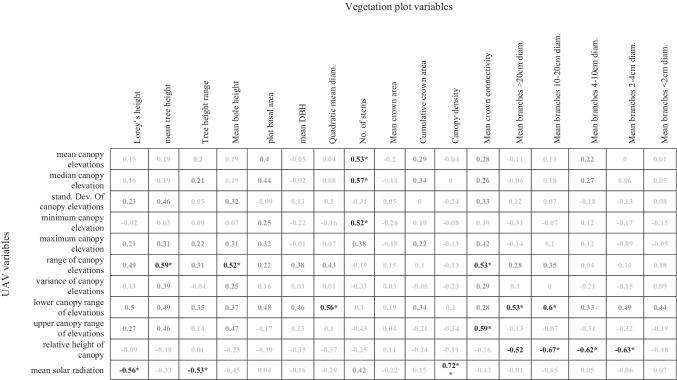
Pearson’s correlation matrix of vegetation plot variables and UAV-derived variables with significantly correlated variables marked with * for *p* < 0.05 or ** for *p* < 0.001, *n* = 15

Additionally, upper canopy range was moderately correlated with tree crown connectivity: ‘mean crown connectivity’ ~ ‘upper canopy range’ (*r* = 0.59, *p* = 0.02). Incoming solar radiation was related to three variables: ‘Lorey’s height’ and ‘mean solar radiation’ (*r* = − 0.56, *p* = 0.03), ‘tree height range’ and ‘mean solar radiation’ (*r* = − 0.53, *p* = 0.040), ‘canopy density’ and ‘mean solar radiation’ (*r* = 0.72, *p* = 0.002). The number of stems within vegetation plots was moderately positively correlated with ‘mean’, ‘median’ and minimum’ elevation, and several categories of branch counts were negatively correlated with ‘relative height’ and ‘lower canopy range’ (see Table 2).

### Boosted regression tree models: vegetation structure effects on the mean and the 95th percentile hourly air temperature

The BRT models performed well in predicting the mean and the 95th percentile hourly microclimatic air temperature and were highly correlated to recorded values (*R*^*2*^ range = 0.899 to 0.959, *p* < 0.001). Percentage of divergence also performed well (*D*^*2*^ range = 0.90 to 0.959) and RSME ranging from 0.596 to 1.12 across models using vegetation plot variables, UAV-derived variables and a combined model using all variables (Table 3). The predicted values from mean hourly microclimatic air temperature models were ~ 5% more accurate than the 95th percentile models as measured by *R*^*2*^ values, 4.86% to 5.34% more accurate as measured by *D*^*2*^ values, and RMSE values were 0.49 to 0.46 more accurate when compared to recorded data. The relative influence of individual variables is shown in Table 3.

**Table 3:**
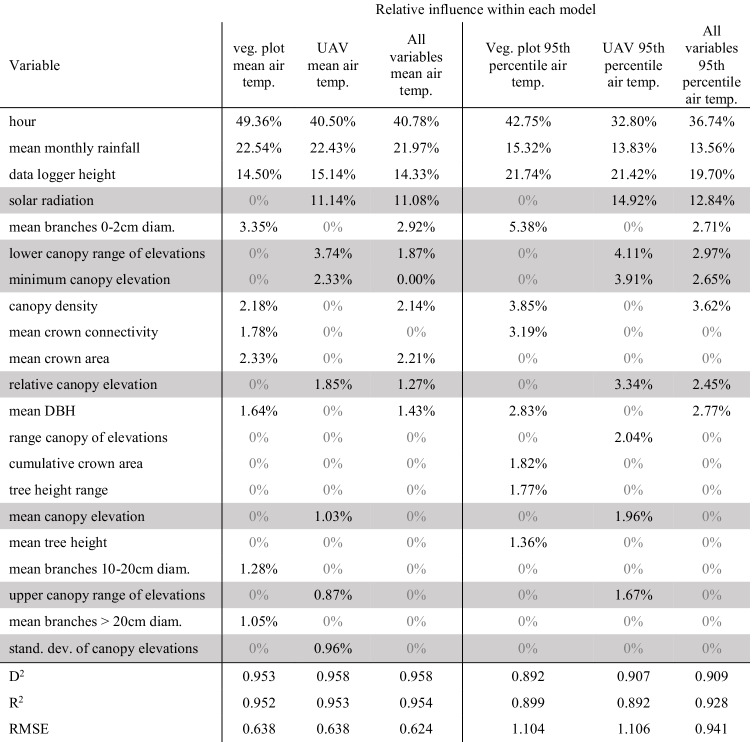
Summary of boosted regression tree models predicting the mean and the 95th percentile hourly microclimatic air temperature, with relative influence of individual vegetation variables on each of three models. Ordered by mean relative influence across models. Variables shaded in grey are derived from UAV surveys

The fitted functions of most response variables in the BRT models are non-linear (Supplementary Materials, Fig. 11a and b). The hour of day response variable, for instance, is the most influential across all BRT models (Table 3) and is largely symmetric, peaking at 2 pm (Supplementary Fig. 11a–b). Data logger height, the third most influential variable across models, is mostly linear in response, with lower values at lower heights, but with notable ‘dips’ in response values at 11–12 m and 21–26 m, possibly due to the canopy being relatively dense at these heights, thus providing extensive shade for the data loggers (Supplementary Fig. 11a–b). Additionally, interactions between variables were strong, particularly between canopy density and incoming solar radiation (for models that included solar radiation and hour for those that did not (Fig. 3, Supplementary Table 1a–f)).

## Discussion

Our study focused on the effects of forest structure on hourly air temperature within Sikundur, a degraded tropical forest in North Sumatra. Our findings showed that Sikundur has a dynamic climatic environment, with differences of up to 15.2 °C in simultaneous measurements of air temperatures within the forest. This is 5 °C more than previously recorded in simultaneous data logger measurements within tropical environments (Scheffers et al. 2014b).). Temperature peaks were highly changeable at different locations across time (Supplementary Fig. 11a–d), suggesting that vegetation structure and topographic location alter the effectiveness of thermal buffering at different times of the day within the canopy (Pringle et al. 2003). It is hard to classify areas of forest as ‘intact’ or ‘degraded’ from the vegetation variables recorded within Sikundur, as the most important indicators of old-growth forest, i.e. high values in mean DBH and basal area, were not significantly different among vegetation plots, unlike previous studies of microclimate within forest areas with defined levels of degradation (Frey et al. 2016; Blonder et al. 2018). Rather, Sikundur exhibits the heterogeneous gradients of degradation which characterise historically logged forest (Struebig et al. 2013). This form of degradation can have a relatively low ecological impact compared with other forms of anthropogenic disturbance, such as fragmentation and fire (Barlow et al. 2006), although historical forest degradation does impact the microclimatic landscape.

The majority of the relative influence of variables within BRT models was not directly related to vegetation structure. The time of day, height of measurement, rainfall and, partially, incoming solar radiation (as it is altered by canopy structure and terrain) had collectively a high relative influence in BRT models (70–86.4%). However, as hour and mean monthly rainfall were equal across all plots, the majority of the variability between sites was determined by measurement height and incoming solar radiation. Each of these variables had strong interactive effects with vegetation variables (Supplementary Table 1a–f). For example, in the hourly mean climatic air temperature model containing all variables, incoming solar radiation was mediated by canopy density, with the most intense solar radiation (350 kWh/m2) within 25 × 25 m vegetation plots with 78% canopy cover having the same impact on mean microclimate air temperature as 75 kWh/m^2^ within vegetation plots with 68% canopy cover (Supplementary Table 1a, Fig. 3). This pattern is repeated for the 95th percentile microclimatic air temperature model, with a stronger interaction (Fig. 3, Supplementary Table 1d), as well as the 95th percentile model using only vegetation plot variables with canopy density and hour of the day (possibly a proxy of the amount of solar radiation) (Fig. 3, Supplementary Table 1f). Whilst these results (Table 3) may seem to diminish the role of vegetation structure in shaping within-canopy microclimatic air temperature, this is likely how microclimates within tropical forests are formed, with temporal effects (time of day, solar radiation) interacting with weather events (rain), and then buffered by vegetation structure (Fig. 3). Additionally, topographical position has a strong effect on the amount of solar radiation reaching a particular location in the canopy (Fig. 2). Shadow effects caused by microtopography (hills, depressions, streams/rivers) and surrounding vegetation structure (including emergent trees) directly impacted one of the primary drivers of microclimatic air temperature: solar radiation (Fig. 2, Table 3). Seemingly, a plot’s location on the landscape (i.e. if it is on a hill on in a valley) has a larger influence on its microclimatic temperature than forest structure, although the two factors have strong interactive effects (Fig. 3).

Plots with high values in variables associated with old-growth forests, such as mean DBH values and mean crown area (Van Pelt and Franklin 2000, Rutishauser et al. 2016; Pereira et al. 2002), directly affected hourly air temperatures (Table 3, Supplementary Fig. 10a–b). Although these impacts were less apparent than abiotic factors in BRT models, variables that measured canopy structure that buffered incoming solar radiation, namely, high canopy density and high branch counts, were still highly influential (Table 3). This suggests that whilst forest age is an important factor in determining microclimate variables (Frey et al. 2016), canopy density, possibly provided by fast-growing vegetation which fills canopy gaps after forest degradation (Chazdon 2003), is a more important factor in determining microclimate air temperature in tropical forests. Plots with high values in canopy density had reduced the mean and the 95th percentile microclimatic air temperature which potentially provides thermal refuges for arboreal organisms. The 95th percentile temperatures were more affected by canopy density and other vegetation variables than mean hourly temperatures, suggesting that in higher temperatures, microclimatic air temperature within forest canopy is increasingly governed by vegetation structure. These results are consistent with the findings of previous studies which were able to define areas of forest by differing levels of anthropogenic disturbance (Jucker et al. 2018; Blonder et al. 2018), though this study shows that historically selectively logged forest can show similar air temperature variability as heavily logged forest (i.e. highly variant simultaneous recordings of air temperature) (Blonder et al. 2018). For organisms that are sensitive to high temperatures, this may render the canopies of selectively logged forest climatically unsuitable during the hottest parts of the day or in a warmer future.

UAV-derived measurements of canopy structure had few definitive relationships with field-based measurements. This is likely due to the inability of UAV-derived structure-from-motion photogrammetry to penetrate the multi-layer canopy of tropical forest (unlike LiDAR), rendering microtopographic variation beneath the canopy, and the structure of the understory, hidden from variables derived from UAV surveys. However, UAV-derived variables explaining canopy elevation range within plots (‘range of canopy elevations’ and ‘lower canopy range of elevations’ and ‘upper canopy range of elevations’) showed strong relationships to plot-level measurements. These variables are related to various measures of mean tree size within plots, including tree height, bole height, quadratic mean diameter, larger branch size counts and crown connectivity, all of which are potentially useful measures of forest structure and age in future studies.

Traditional measures of forest age (i.e. high values in mean DBH, mean crown area, plot basal area, branch counts of various sizes) accounted for a significant proportion of relative influence in the BRT models using only vegetation plot variables (13.6, 20%) and have been repeatedly shown to influence the microclimatic environment (Frey et al. 2016; Chen et al. 1995; Parker et al. 2004). However, microclimatic air temperature was marginally better predicted by UAV measurements of canopy structure. The ability of UAVs to provide wide-ranging, detailed measures of canopy surface topography, as well as the estimation of incoming solar radiation (which is the primary driver of air temperature) (Bramer et al. 2018), may be a useful tool in future studies. UAV-derived DSMs are effectively a coarse-grained abstraction for many variables usually measured in microclimate studies (slope, aspect, relative topographic position, vegetation, wind exposure) (Dobrowski 2011). Whilst UAV-derived variables may not account for fine-grained fluctuations in microtopography and other influences on microclimate (e.g. presence of streams or pools, soil moisture, understory structure), it does allow for rapid assessment of these combined effects on microclimatic air temperature at a scale relevant to most organisms. This suggests that UAV surveys, with the addition of in situ microclimate measurements, are able to predict microclimatic air temperature at a given height and time of day within dense forest. Using spatial predictions of the BRT model presented here, it is possible to project mean hourly air temperature across the 25.7 km^2^ cover by UAV surveys (Supplementary Materials, Fig. 12).

## Conclusions

Coupling detailed plot-level vegetation surveys, UAV canopy topography data and microclimate data loggers within the tree canopy has shown a relationship between vegetation structure and microclimatic air temperature at a scale relevant to tropical species. As global temperatures increase, the importance of canopy density and old-growth forest structures in buffering arboreal organisms from extreme temperatures may therefore also increase. Forest degradation limits the buffering capacity of forests. In the face of predicted future climate change, where temperatures are predicted to rise and precipitation levels to become more spatially and temporarily erratic (Tangang et al. 2017), degraded tropical forest may become less suitable for many organisms that have historically inhabited these areas. The use of wide-ranging UAV canopy surveys and data loggers to model air temperature over large areas allows researchers and forest managers to explore the effects of future climate change within canopies over large areas and, in turn, predict the effects of these changes on species dependent on these habitats.

## Supplementary Information

Below is the link to the electronic supplementary material.

Supplementary file1 (DOCX 56699 kb)

## References

[CR1] Abdulhadi R, Mirmanto E, Kartawinata K (1987) A lowland dipterocarp forest in Sekundur, North Sumatra, Indonesia: five years after mechanized logging. In: Proceedings of the Third Round Table Conference on Dipterocarps, ed Kostermans AJGH (pp. 255–273).

[CR2] Alexander C, Korstjens AH, Hankinson E, Usher G, Harrison N, Nowak MG, Abdullah A, Wich S, Hill RA, R. A.  (2018). Locating emergent trees in a tropical rainforest using data from an unmanned aerial vehicle (UAV). International Journal of Applied Earth Observation Geoinformation.

[CR3] Alexander C, Korstjens AH, Hill RA (2017). Structural attributes of individual trees for identifying homogeneous patches in a tropical rainforest. International Journal of Applied Earth Observation Geoinformation.

[CR4] Anderson K, Gaston KJ (2013). Lightweight unmanned aerial vehicles will revolutionize spatial ecology. Front Ecol Environ.

[CR5] Arroyo-Rodríguez V, Saldaña-Vázquez RA, Fahrig L, Santos BA (2016). Does forest fragmentation cause an increase in forest temperature?. Ecol Res.

[CR6] Askew JA, Morrogh-Bernard HC (2016). Acoustic characteristics of long calls produced by male orang-utans (*Pongo pygmaeus wurmbii*): advertising individual identity, context, and travel direction. Folia Primatol.

[CR7] Barlow J, Peres CA, Henriques LMP, Stouffer PC, Wunderle JM (2006). The responses of understorey birds to forest fragmentation, logging and wildfires: an Amazonian synthesis. Biol Cons.

[CR8] Blonder B, Both S, Coomes DA, Elias D, Jucker T, Kvasnica J, Svátek M (2018). Extreme and highly heterogeneous microclimates in selectively logged tropical forests. Frontiers in Forests and Global Change.

[CR9] Bramer I, Anderson BJ, Bennie J, Bladon AJ, De Frenne P, Hemming D, Hill RA, Kearney MR, Korner C, Korstjens AH, Lenoir J, Maclean IMD, Marsh CD, Morecroft MD, Ohlemuller R, Slater HD, Suggitt AJ, Zellweger F, Gillingham P K (2018) Advances in monitoring and modelling climate at ecologically relevant scales. In Advances in ecological research (Vol. 58, pp. 101–161). Academic Press.

[CR10] Brock FV, Richardson J, Semmer SR (1995) Passive multiplate solar radiation shields. In Preprints, Ninth Symp. on Meteorological Observations and Instrumentation, Charlotte, NC, Amer. Meteor. Soc (pp. 329–334)

[CR11] Brokaw NW (1982). The definition of treefall gap and its effect on measures of forest dynamics. Biotropica.

[CR12] Chazdon RL (2003). Tropical forest recovery: legacies of human impact and natural disturbances. Perspectives in Plant Ecology, Evolution and Systematics.

[CR13] Chen J, Franklin JF, Spies TA (1995). Growing-season microclimatic gradients from clearcut edges into old-growth Douglas-fir forests. Ecol Appl.

[CR14] Chen J, Saunders SC, Crow TR, Naiman RJ, Brosofske KD, Mroz GD, Franklin JF (1999). Microclimate in forest ecosystem and landscape ecology: variations in local climate can be used to monitor and compare the effects of different management regimes. Bioscience.

[CR15] Conrad O, Bechtel B, Bock M, Dietrich H, Fischer E, Gerlitz L, Wehberg J, Wichmann V, Böhner J (2015). System for automated geoscientific analyses (SAGA) v. 2.1.4. Geosciences Model Devision.

[CR16] Dobrowski SZ (2011). A climatic basis for microrefugia: the influence of terrain on climate. Glob Change Biol.

[CR17] Dray S, Saïd S, Débias F (2008). Spatial ordination of vegetation data using a generalization of Wartenberg’s multivariate spatial correlation. J Veg Sci.

[CR18] Eckmann T, Morach A, Hamilton M, Walker J, Simpson L, Lower S, ... & Kessi A (2018) Measuring and modelling microclimate impacts of Sequoiadendron giganteum. Sustainable cities and society, 38, 509-525

[CR19] Fick SE, Hijmans RJ (2017). WorldClim 2: new 1-km spatial resolution climate surfaces for global land areas. Int J Climatol.

[CR20] Frey SJ, Hadley AS, Johnson SL, Schulze M, Jones JA, Betts MG (2016) Spatial models reveal the microclimatic buffering capacity of old-growth forests. Science Advances, 2(4), e1501392. 10.1126/sciadv.1501392 PMC4846426 27152339

[CR21] Ganzhorn JU, Jacques Rakotondranary S, Ratovonamana YR, Setchell JM, Curtis DJ (2011). Habitat description and phenology. Field and laboratory methods in primatology: a practical guide.

[CR22] González del Pliego P, Scheffers BR, Basham EW, Woodcock P, Wheeler C, Gilroy JJ, Medina Uribe CA, Haugaasen T, Freckleton RP, Edwards D (2016). Thermally buffered microhabitats recovery in tropical secondary forests following land abandonment. Biology Conservation.

[CR23] Goodenough AE, Goodenough AS (2012). Development of a rapid and precise method of digital image analysis to quantify canopy density and structural complexity. ISRN Ecology.

[CR24] Graae, B. J., De Frenne, P., Kolb, A., Brunet, J., Chabrerie, O., Verheyen, K., ... & Nijs, I. (2012). On the use of weather data in ecological studies along altitudinal and latitudinal gradients. Oikos, 121(1), 3-19

[CR25] Hankinson EL, Hill RA, Marsh CD, Nowak MG, Abdullah A, Pasaribu N, Supriadi, Nijman V, Cheyne SM, Korstjens AH (2021) Influences of forest structure on the density and habitat preference of two sympatric gibbons (Symphalangus syndactylus and Hylobates lar). International Journal of Primatology, 42, 237–261

[CR26] Harper KA, Macdonald SE, Burton PJ, Chen J, Brosofske KD, Saunders SC, Esseen PA (2005). Edge influence on forest structure and composition in fragmented landscapes. Conserv Biol.

[CR27] Harrison NJ, Hill RA, Alexander C, Marsh CD, Nowak MG, Abdullah A, Slater HD, Korstjens AH (2020) Sleeping trees and sleep-related behaviours of the siamang (Symphalangus syndactylus) in a tropical lowland rainforest, Sumatra, Indonesia. Primates, 1–13 10.1007/s10329-020-00849-8 PMC7813730 32720108

[CR28] Hill RA, Hinsley SA (2015). Airborne lidar for woodland habitat quality monitoring: Exploring the significance of lidar data characteristics when modelling organism-habitat relationships. Remote Sensing.

[CR29] Hiscocks P (2011) Measuring light. Ryerson University.

[CR30] Hubbart J, Link T, Campbell C, Cobos D (2005). Evaluation of a low-cost temperature measurement system for environmental applications. Hydrological Processes: an International Journal.

[CR31] Hummel S, Hudak AT, Uebler EH, Falkowski MJ, Megown KA (2011). A comparison of accuracy and cost of LiDAR versus stand exam data for landscape management on the Malheur National Forest. J Forest.

[CR32] Jenkins DG, Brescacin CR, Duxbury CV, Elliott JA, Evans JA, Grablow KR, Pepe D (2007). Does size matter for dispersal distance?. Glob Ecol Biogeogr.

[CR33] Jones HG (2013). Plants and microclimate: a quantitative approach to environmental plant physiology.

[CR34] Jucker T, Hardwick SR, Both S, Elias DM, Ewers RM, Milodowski DT, Coomes DA (2018). Canopy structure and topography jointly constrain the microclimate of human-modified tropical landscapes. Glob Change Biol.

[CR35] Kays R, Allison A (2001) Arboreal tropical forest vertebrates: current knowledge and research trends. pp. 109–120, In: Tropical Forest Canopies: Ecology and Management, Springer, Dordrecht

[CR36] Kearney MR, Porter WP (2017). NicheMapR–an R package for biophysical modelling: the microclimate model. Ecography.

[CR37] Knop E, Ward PI, Wich SA (2004). A comparison of orang-utan density in a logged and unlogged forest on Sumatra. Biol Cons.

[CR38] Koh LP, Wich SA (2012). Dawn of drone ecology: low-cost autonomous aerial vehicles for conservation. Tropical Conservation Science.

[CR39] Laurance WF, Nascimento HE, Laurance SG, Andrade AC, Fearnside PM, Ribeiro JE, Capretz RL (2006). Rain forest fragmentation and the proliferation of successional trees. Ecology.

[CR40] Lisein J, Pierrot-Deseilligny M, Bonnet S, Lejeune P (2013). A photogrammetric workflow for the creation of a forest canopy height model from small unmanned aerial vehicle imagery. Forests.

[CR41] Malhi Y, Wright J (2004). Spatial patterns and recent trends in the climate of tropical rainforest regions. Philosophical Transactions of the Royal Society of London b: Biological Sciences.

[CR42] Malhi Y, Aragão LE, Galbraith D, Huntingford C, Fisher R, Zelazowski P, Meir P (2009). Exploring the likelihood and mechanism of a climate-change-induced dieback of the Amazon rainforest. Proc Natl Acad Sci.

[CR43] Manduell KL, Harrison ME, Thorpe SK (2012). Forest structure and support availability influence orang-utan locomotion in Sumatra and Borneo. Am J Primatol.

[CR44] Mulkey SS, Pearcy RW (1992). Interactions between acclimation and photoinhibitation of photosynthesis of a tropical forest understory herb, Alocasia macrorrhiza, during simulate canopy gap formation. Funct Ecol.

[CR45] Norris C, Hobson P, Ibisch PL (2012). Microclimate and vegetation function as indicators of forest thermodynamic efficiency. J Appl Ecol.

[CR46] Nowakowski AJ, Frishkoff LO, Agha M, Todd BD, Scheffers BR (2018). Changing thermal landscapes: merging climate science and landscape ecology through thermal biology. Current Landscape Ecology Reports.

[CR47] Oliver TH, Morecroft MD (2014). Interactions between climate change and land use change on biodiversity: attribution problems, risks, and opportunities. Wiley Interdisciplinary Reviews: Climate Change.

[CR48] Parker GG, Harmon ME, Lefsky MA, Chen J, Van Pelt R, Weis SB, ... & Frankling JF (2004) Three-dimensional structure of an old-growth Pseudotsuga-Tsuga canopy and its implications for radiation balance, microclimate, and gas exchange. Ecosystems, 7(5), 440-453

[CR49] Pereira R, Zweede J, Asner GP, Keller M (2002). Forest canopy damage and recovery in reduced-impact and conventional selective logging in eastern Para, Brazil. Forest Ecology and Management.

[CR50] Pohlman CL, Turton SM, Goosem M (2007). Edge effects of linear canopy openings on tropical rain forest understory microclimate. Biotropica.

[CR51] Potter KM, Hargrove WW (2013) Quantitative metrics for assessing predicted climate change pressure on North American tree species. International Journal of Mathematical Computational Forestry and Nature Reseach Sciences 5 (2): 151–169, 5(2), 151–169

[CR52] Potzger JE (1939). Microclimate and a notable case of its influence on a ridge in central Indiana. Ecology.

[CR53] Pringle RM, Webb JK, Shine R (2003). Canopy structure, microclimate, and habitat selection by a nocturnal snake. Hoplocephalus Bungaroides Ecology.

[CR54] R Core Team (2019). R: A language and environment for statistical computing. R Foundation for Statistical Computing, Vienna, Austria. ISBN 3–900051–07–0, URL http://www.R-project.org/.

[CR55] Richardson SJ, Brock FV, Semmer SR, Jirak C (1999). Minimizing errors associated with multiplate radiation shields. J Atmos Oceanic Tech.

[CR56] Rutishauser E, Hérault B, Petronelli P, Sist P (2016). Tree height reduction after selective logging in a tropical forest. Biotropica.

[CR57] Senior RA, Hill JK, González del Pliego P, Goode LK, Edwards DP (2017). A pantropical analysis of the impacts of forest degradation and conversion on local temperature. Ecol Evol.

[CR58] Scheffers BR, Edwards DP, Diesmos A, Williams SE, Evans TA (2014). Microhabitats reduce animal’s exposure to climate extremes. Glob Change Biol.

[CR59] Scheffers BR, Evans TA, Williams SE, Edwards DP (2014). Microhabitats in the tropics buffer temperature in a globally coherent manner. Biol Let.

[CR60] Scheffers BR, Shoo L, Phillips B, Macdonald SL, Anderson A, VanDerWal J, Williams SE (2017). Vertical (arboreality) and horizontal (dispersal) movement increase the resilience of vertebrates to climatic instability. Glob Ecol Biogeogr.

[CR61] Sirami C, Caplat P, Popy S, Clamens A, Arlettaz R, Jiguet F, Martin JL (2017). Impacts of global change on species distributions: obstacles and solutions to integrate climate and land use. Glob Ecol Biogeogr.

[CR62] Struebig MJ, Turner A, Giles E, Lasmana F, Tollington S, Bernard H, Bell D (2013) Quantifying the biodiversity value of repeatedly logged rainforests: gradient and comparative approaches from Borneo. In Advances in ecological research (Vol. 48, pp. 183–224). Academic Press

[CR63] Tangang F, Juneng L, Aldrian E (2017). Observed changes in extreme temperature and precipitation over Indonesia. Int J Climatol.

[CR64] Todd BD, Andrews KM (2008). (2008) Response of a reptile guild to forest harvesting. Conserv Biol.

[CR65] Tuff KT, Tuff T, Davies KF (2016). A framework for integrating thermal biology into fragmentation research. Ecol Lett.

[CR66] van Lierop P, Lindquist E, Sathyapala S, Franceschini G (2015). Global forest area disturbance from fire, insect pests, diseases and severe weather events. For Ecol Manage.

[CR67] Van Pelt R, Franklin JF (2000). Influence of canopy structure on the understory environment in tall, old-growth, conifer forests. Can J for Res.

[CR68] WallisDeVries MF, Baxter W, Van Vliet AJ (2011). Beyond climate envelopes: effects of weather on regional population trends in butterflies. Oecologia.

[CR69] Wang S, Fu B, Gao G, Liu Y, Zhou J (2013). Responses of soil moisture in different land cover types to rainfall events in a re-vegetation catchment area of the Loess Plateau, China. CATENA.

[CR70] Wich S, Dellatore D, Houghton M, Ardi R, Koh LP (2015). A preliminary assessment of using conservation drones for Sumatran orang-utan (*Pongo abelii*) distribution and density. Journal of Unmanned Vehicle Systems.

[CR71] Zahawi RA, Dandois JP, Holl KD, Nadwodny D, Reid JL, Ellis EC (2015). Using lightweight unmanned aerial vehicles to monitor tropical forest recovery. Biol Cons.

[CR72] Zellweger F, De Frenne P, Lenoir J, Rocchini D, Coomes D (2019). Advances in microclimate ecology arising from remote sensing. Trends Ecol Evol.

