# Supplementary material for: Measuring and modelling microclimatic air temperature in a historically degraded tropical forest
Source: Int J Biometeorol. 2022 Mar 31;66(6):1283–95. doi: 10.1007/s00484-022-02276-4 (PMC9132844; doi:10.1007/s00484-022-02276-4)
Supplement: Supplementary file 1 — Supplementary file1 (DOCX 56699 kb) [file 484_2022_2276_MOESM1_ESM.docx]

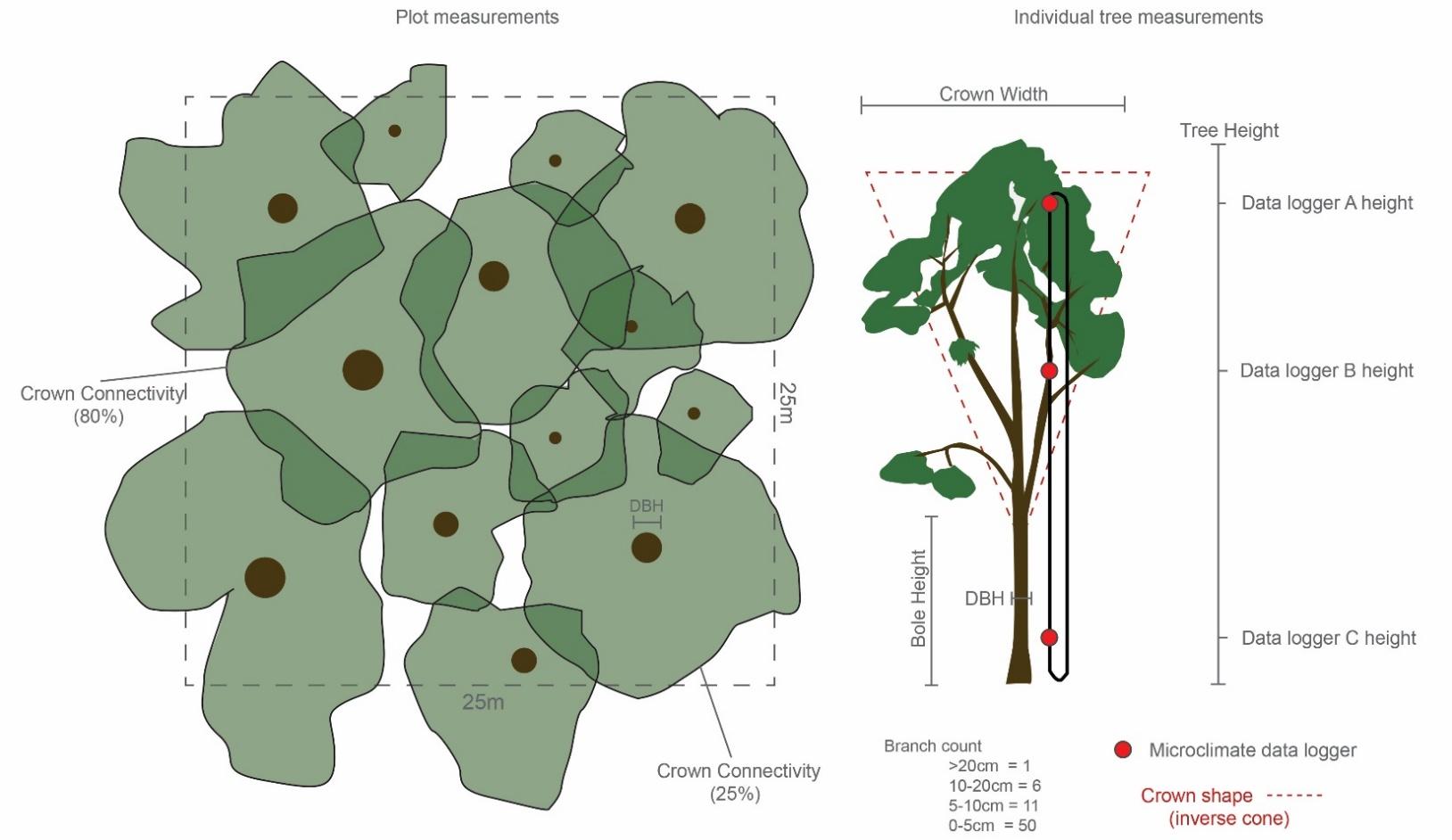
Supplementary materials

Figure 1. Illustrative diagram of vegetation plot variables measured

Figure 2. **A**. Top down view of modified the “Skywalker” fixed-wing UAV used with major parts labelled **B.** Side view of the UAV with major parts labelled **C.** Illustrative wiring diagram of components ^1^ HK Pilot Analog Air Speed Sensor And Pitot Tube Set ^2^ RFD 800 - 900mhz Ultra long-range radio modem ^3^ Micro HKPilot Telemetry with Integrated PCB Antenna 915Mhz ^4^ E-Switch safety switch button (inc. w/Fixhawk) ^5^ SunnySky 2820 800 kv ^6^ TURNIGY Plush 60amp Speed Controller ^8^ Fixhawk autopilot ^9^ Seagull #MAP 2 ^10^ 30mm Buzzer (inc. w/Fixhawk) ^11^ UBLOX Micro M8N GPS Compass Module ^12^ RFDANT 900 receiver^13^ Sony RX100 mk4 ^14^ Flex1 902-928 MHz ISM Band Antenna ^15^ Multistar High Capacity 10000mAh 4S 12C LipPo Pack ^16^ Futaba T8J 8 Channel Digital Proportional Radio Control System


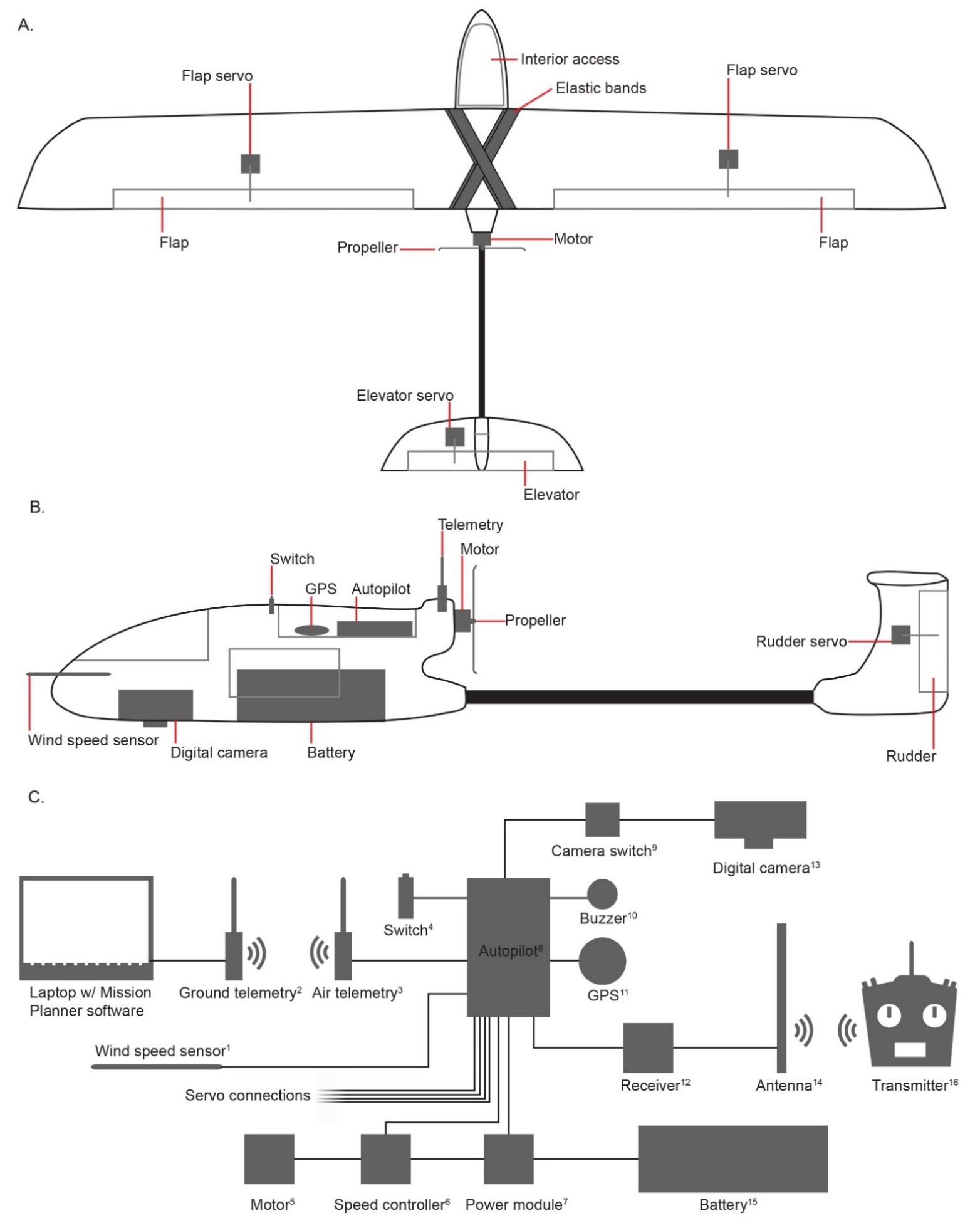


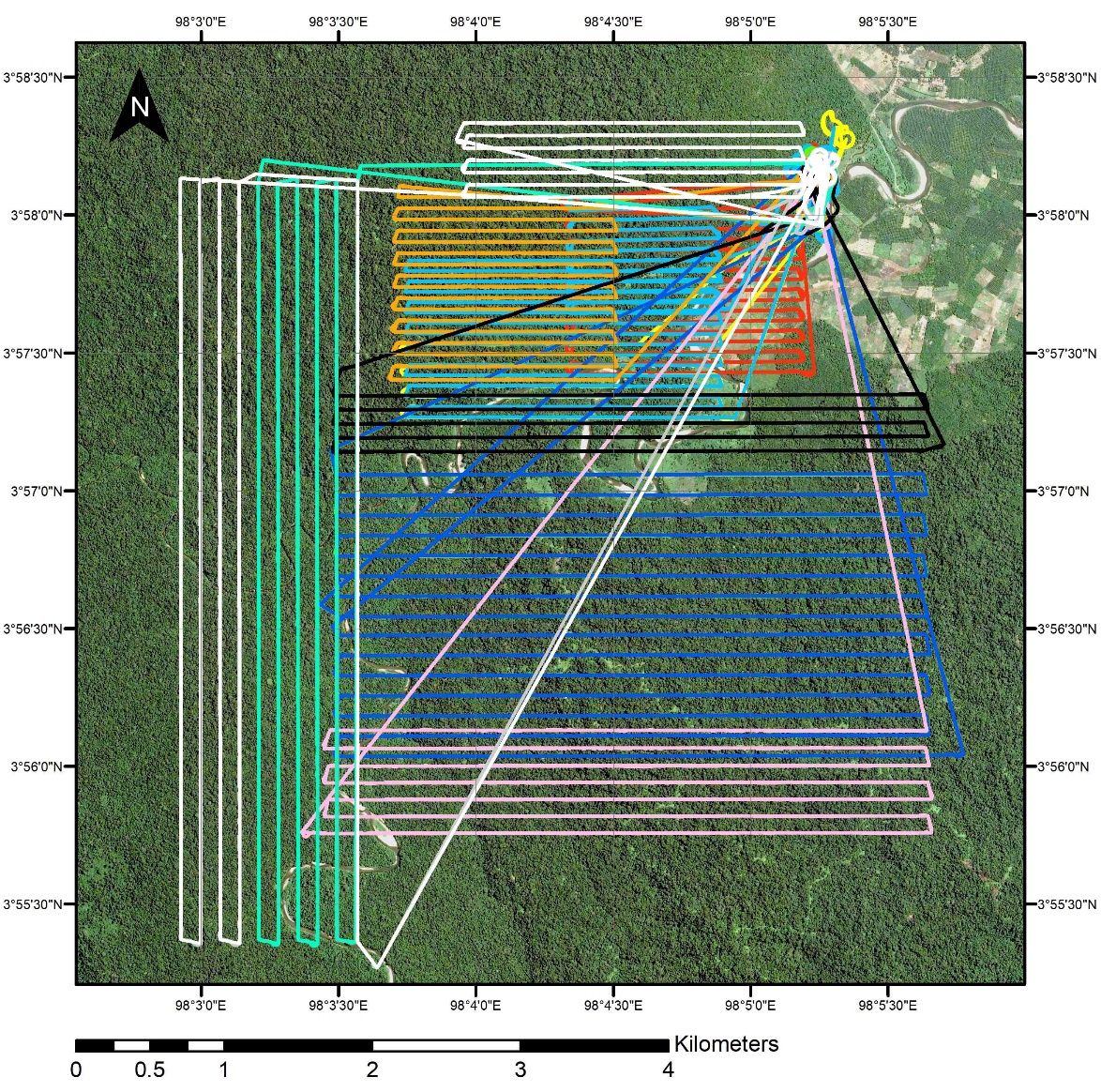


Figure 3. UAV flight paths over the Sikundur area with each flight shown in a separate colour


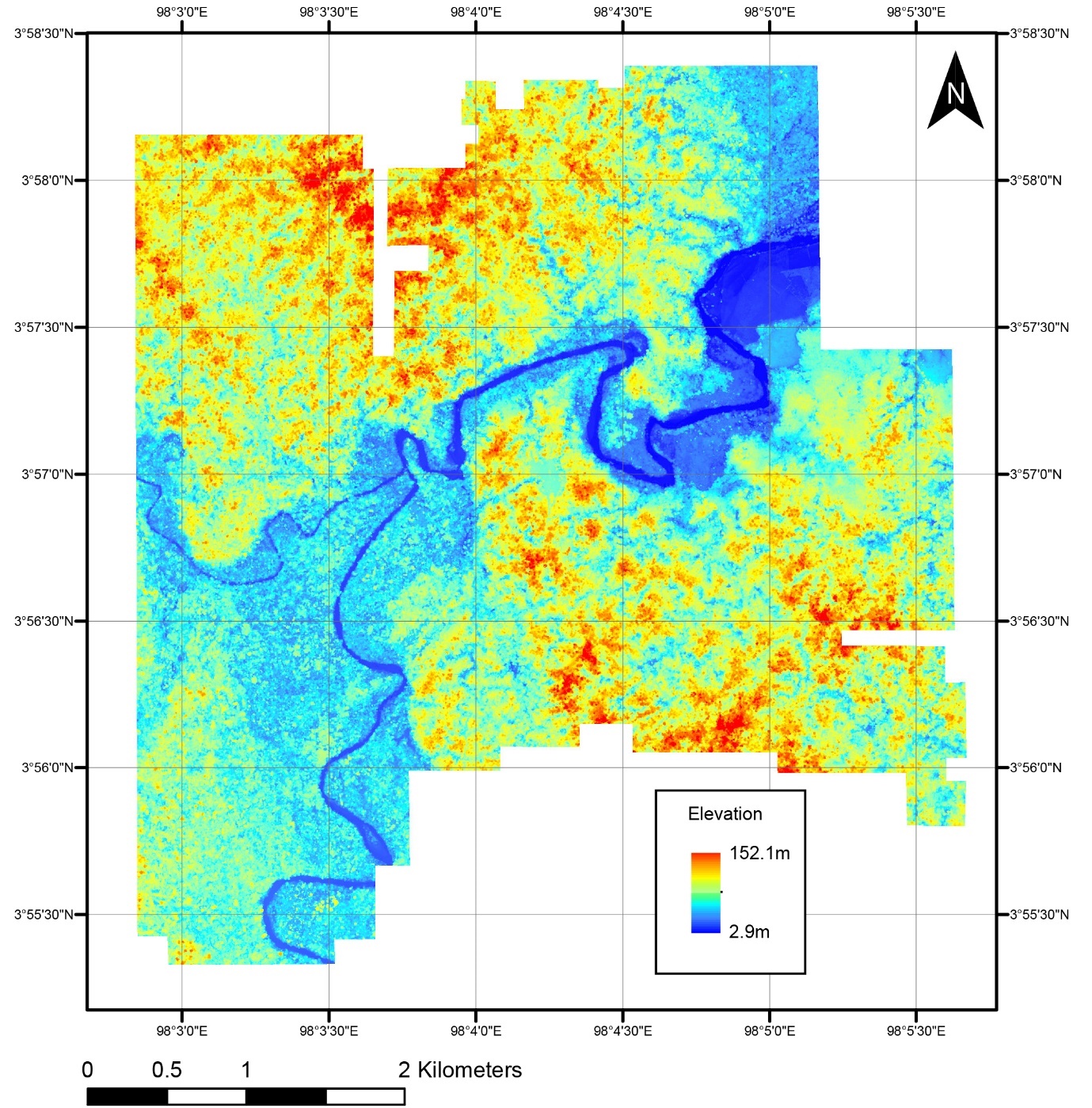


Figure 4. Resulting Digital Surface Model with areas of low accuracy removed


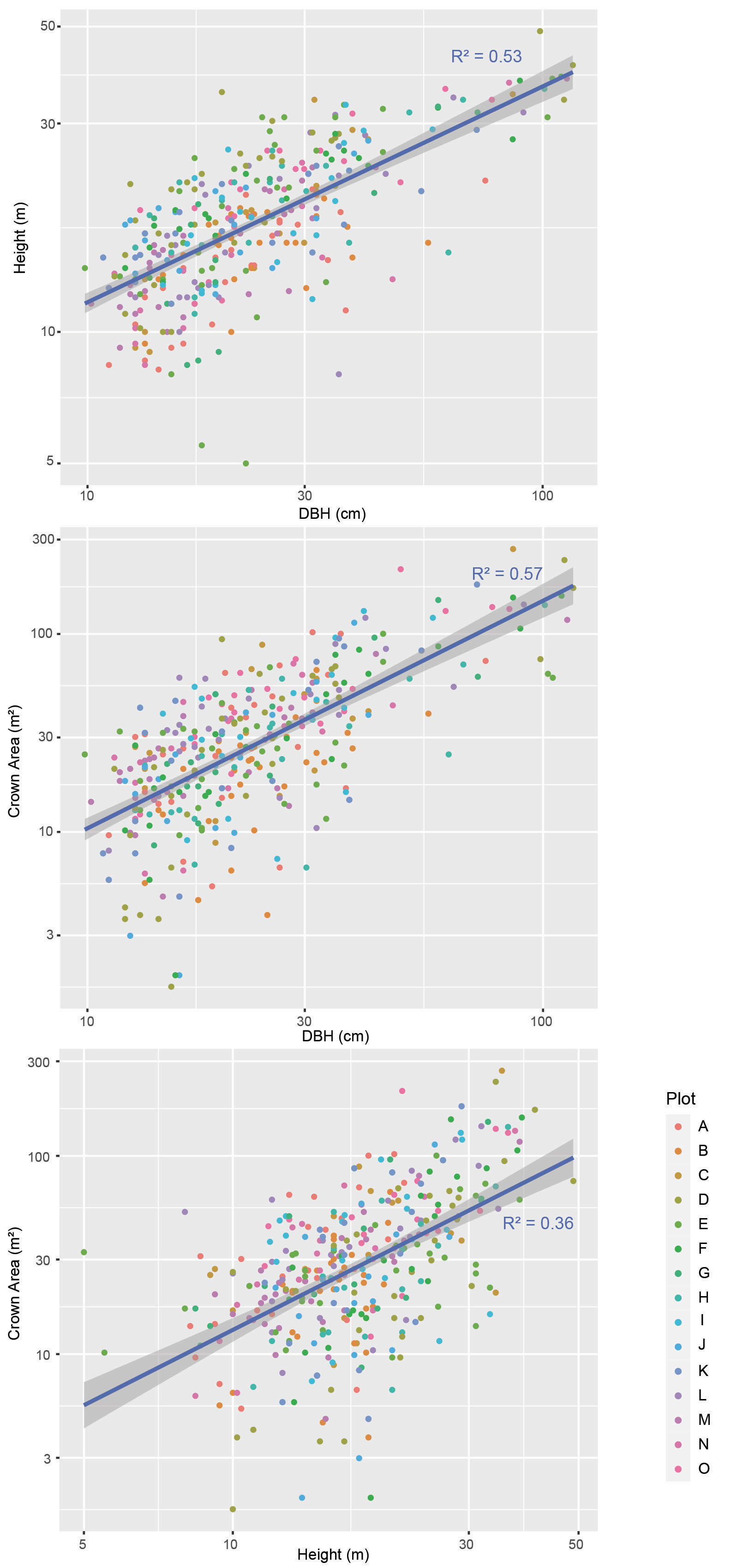


Figure 5. Linear relationships between vegetation variables as measured in vegetation plots displayed on a logarithmic scale


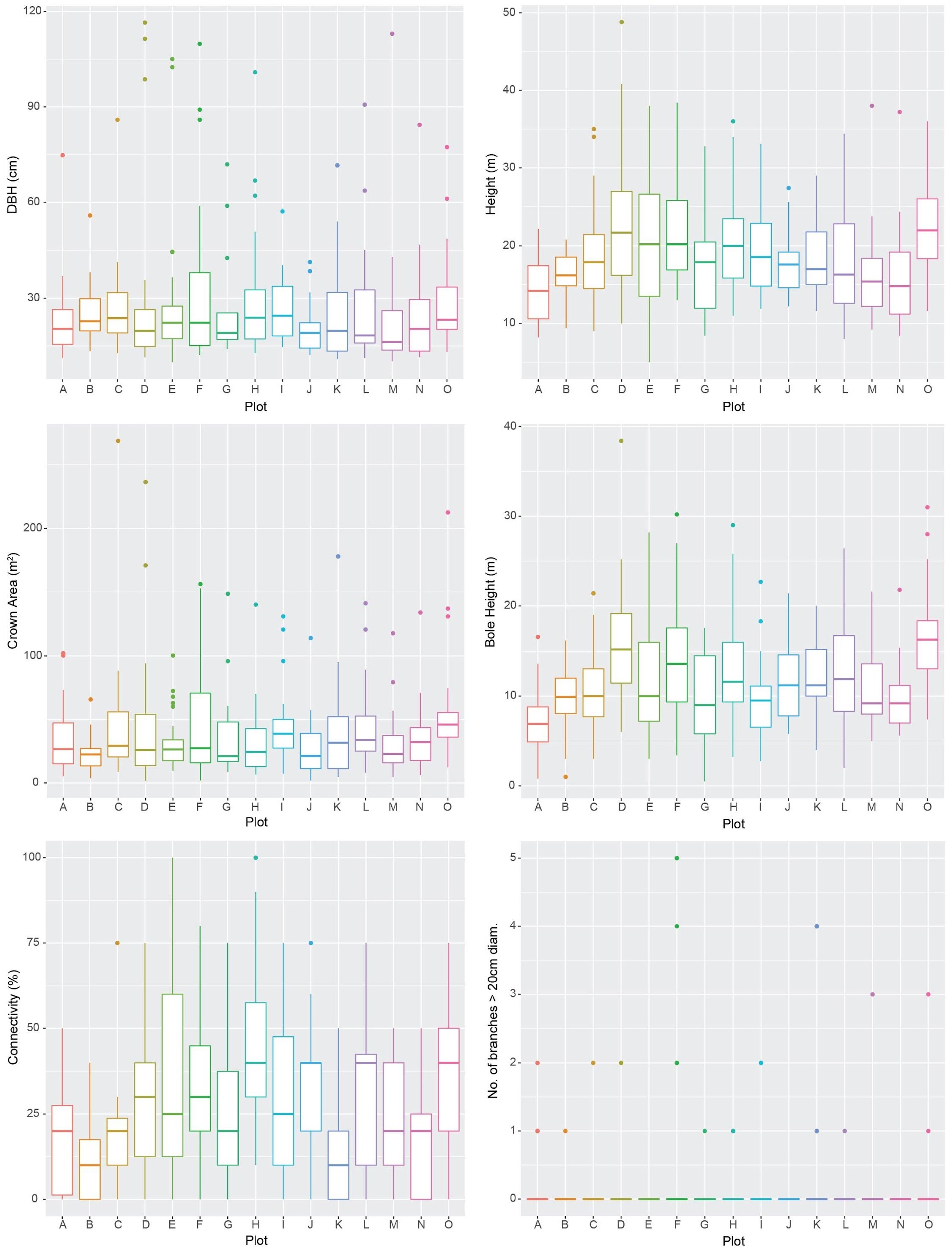
Figure 6. Vegetation plots variable values


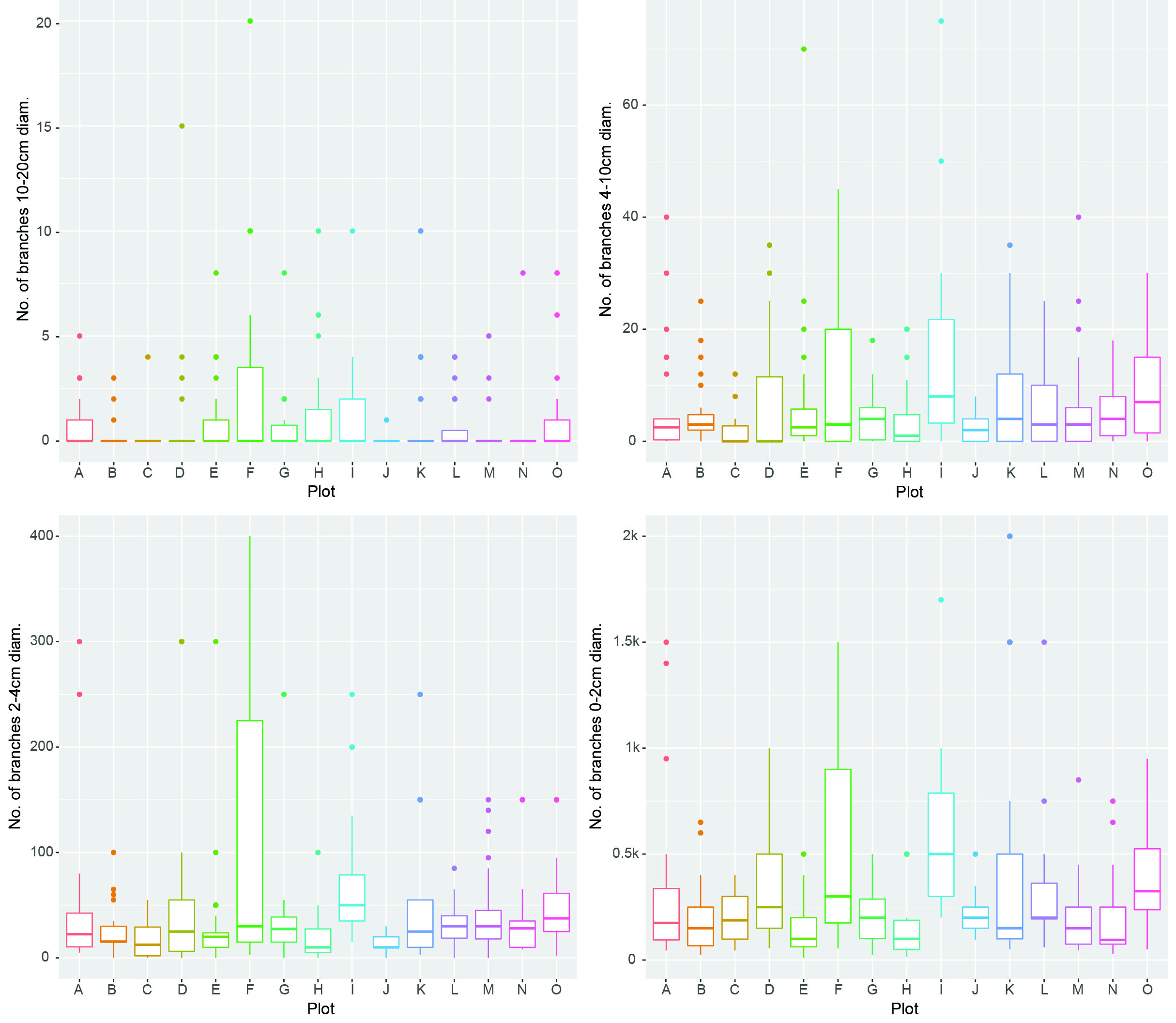
Figure 7. Vegetation plot variables values


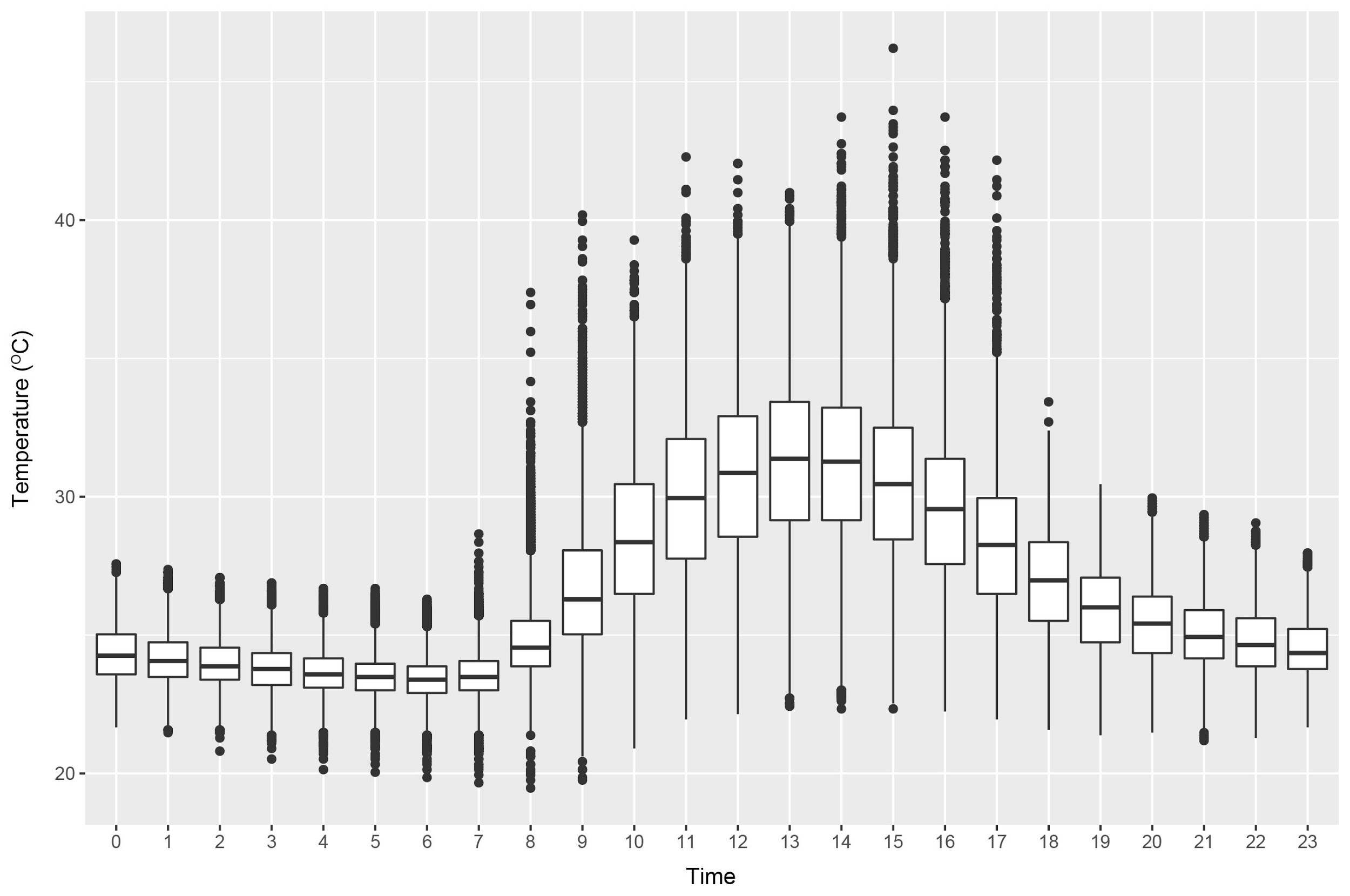

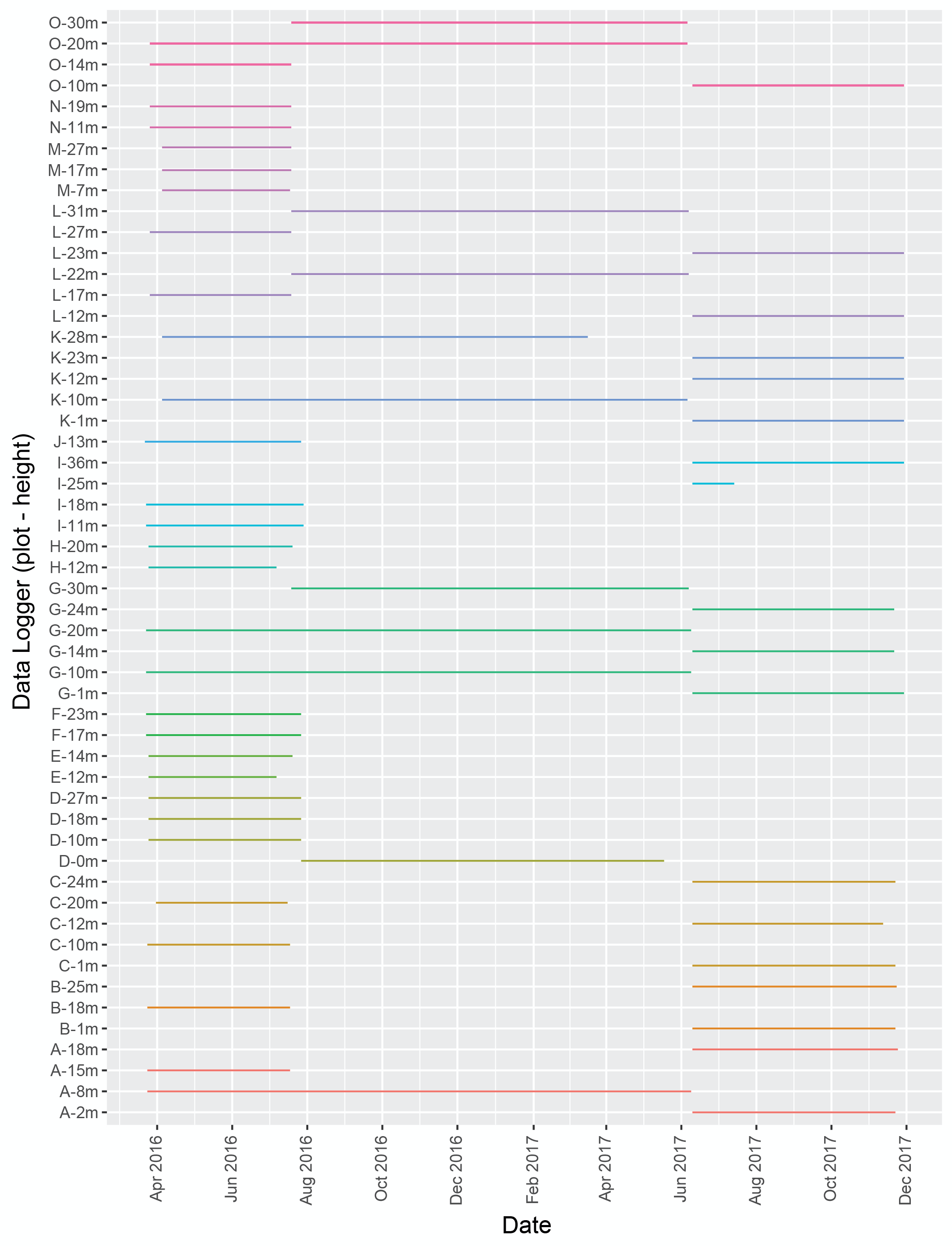
Figure 8. All microclimatic air temperature recordings, across all heights and locations, displayed by the hour of the day
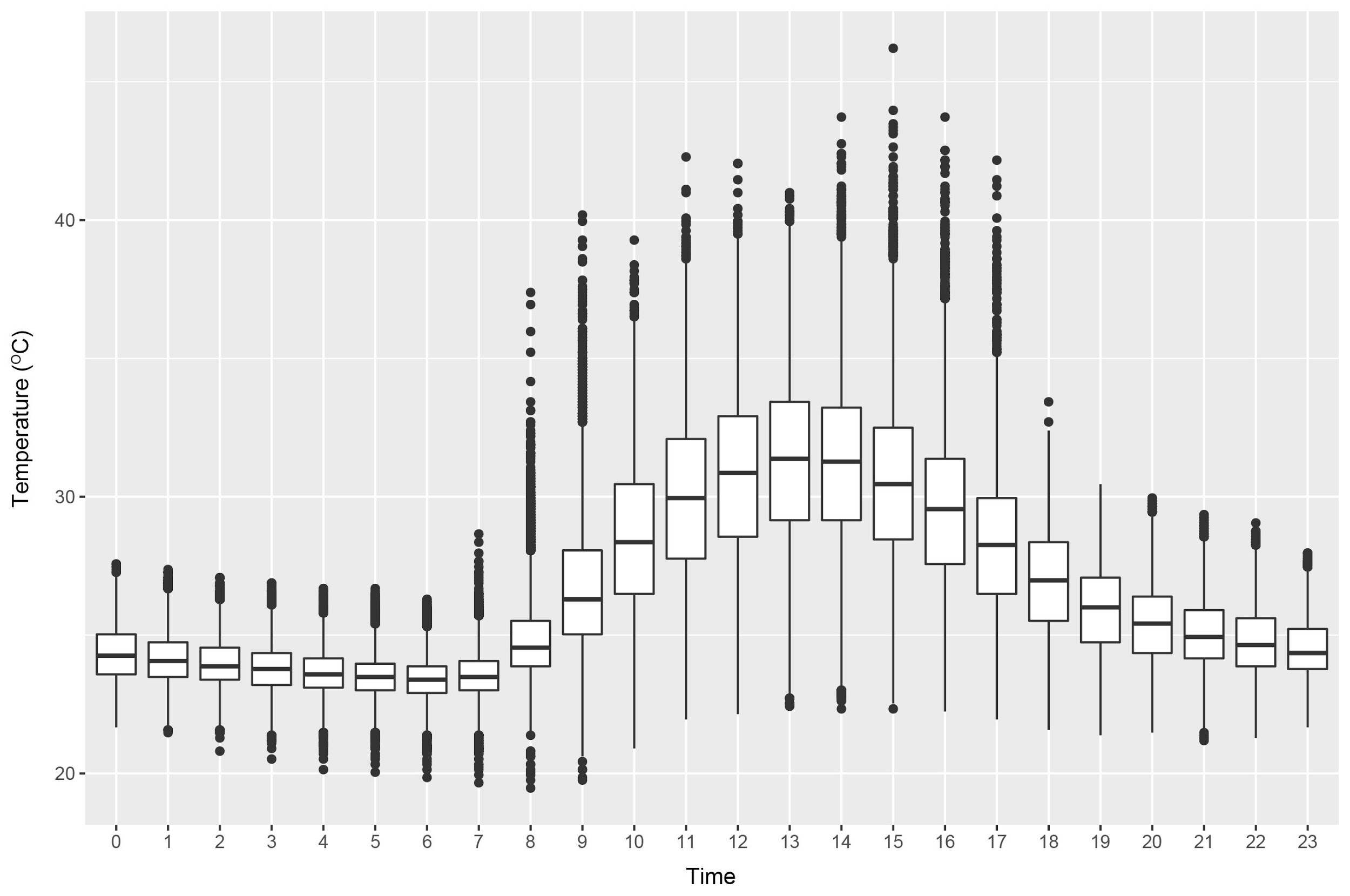


Figure 9. Operating times of data loggers throughout the study period


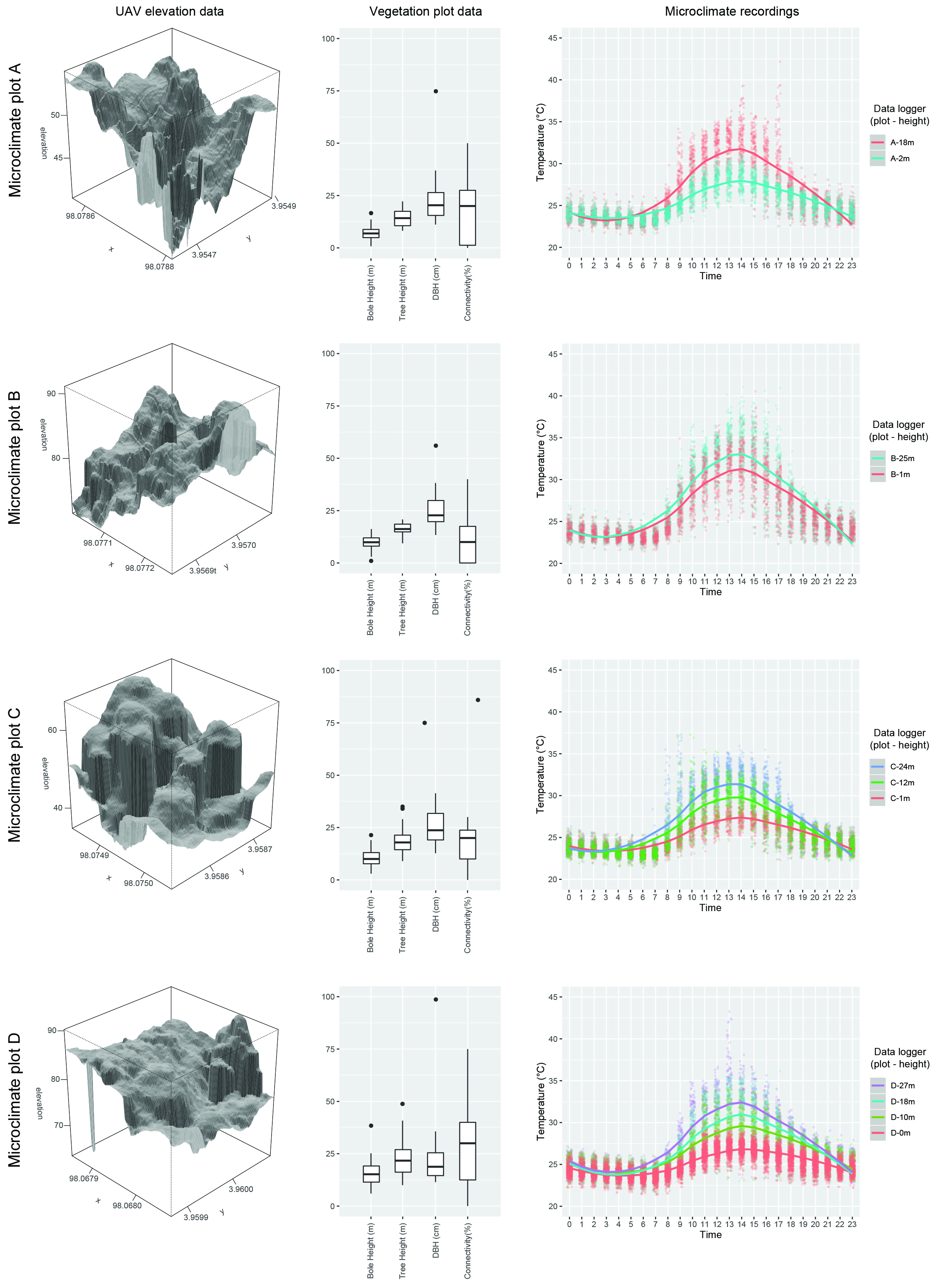
Figure 10a. Each plot’s UAV derived canopy topography, a sample of vegetation plot variables and temperatures recorded by dataloggers operating on the same dates


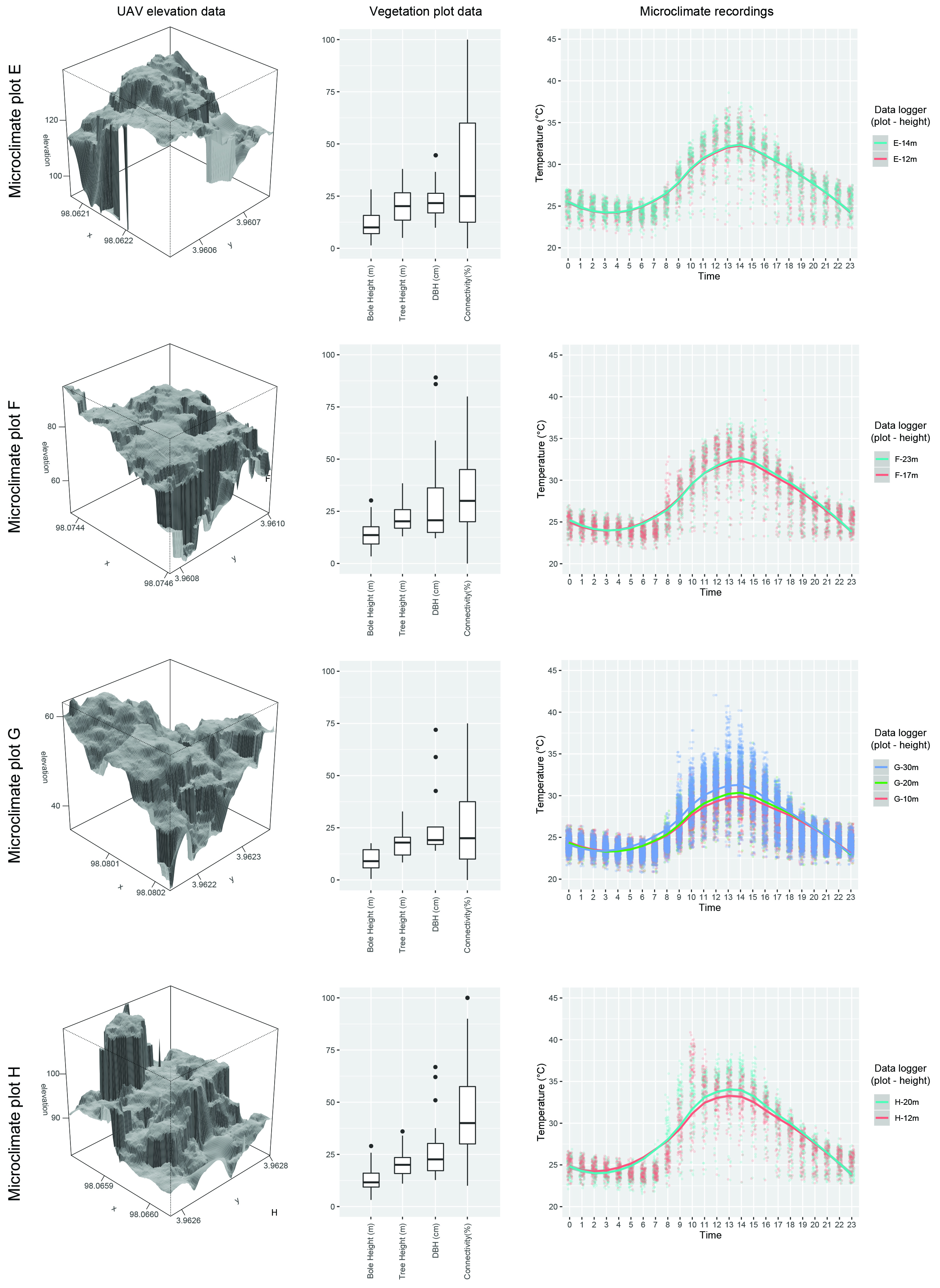
Figure 10b. Each plot’s UAV derived canopy topography, a sample of vegetation plot variables and temperatures recorded by dataloggers operating on the same dates


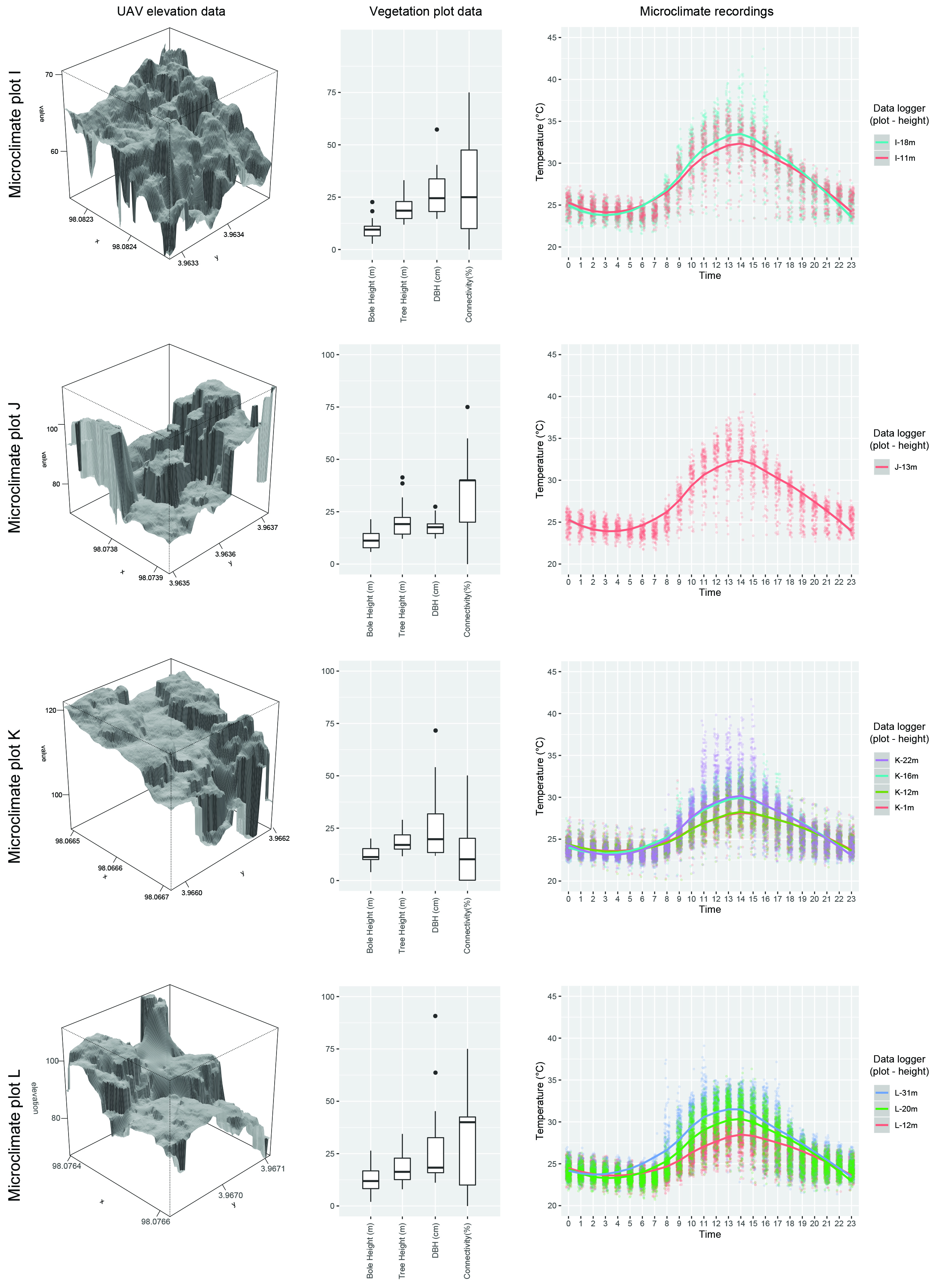
Figure 10c. Each plot’s UAV derived canopy topography, a sample of vegetation plot variables and temperatures recorded by dataloggers operating on the same dates


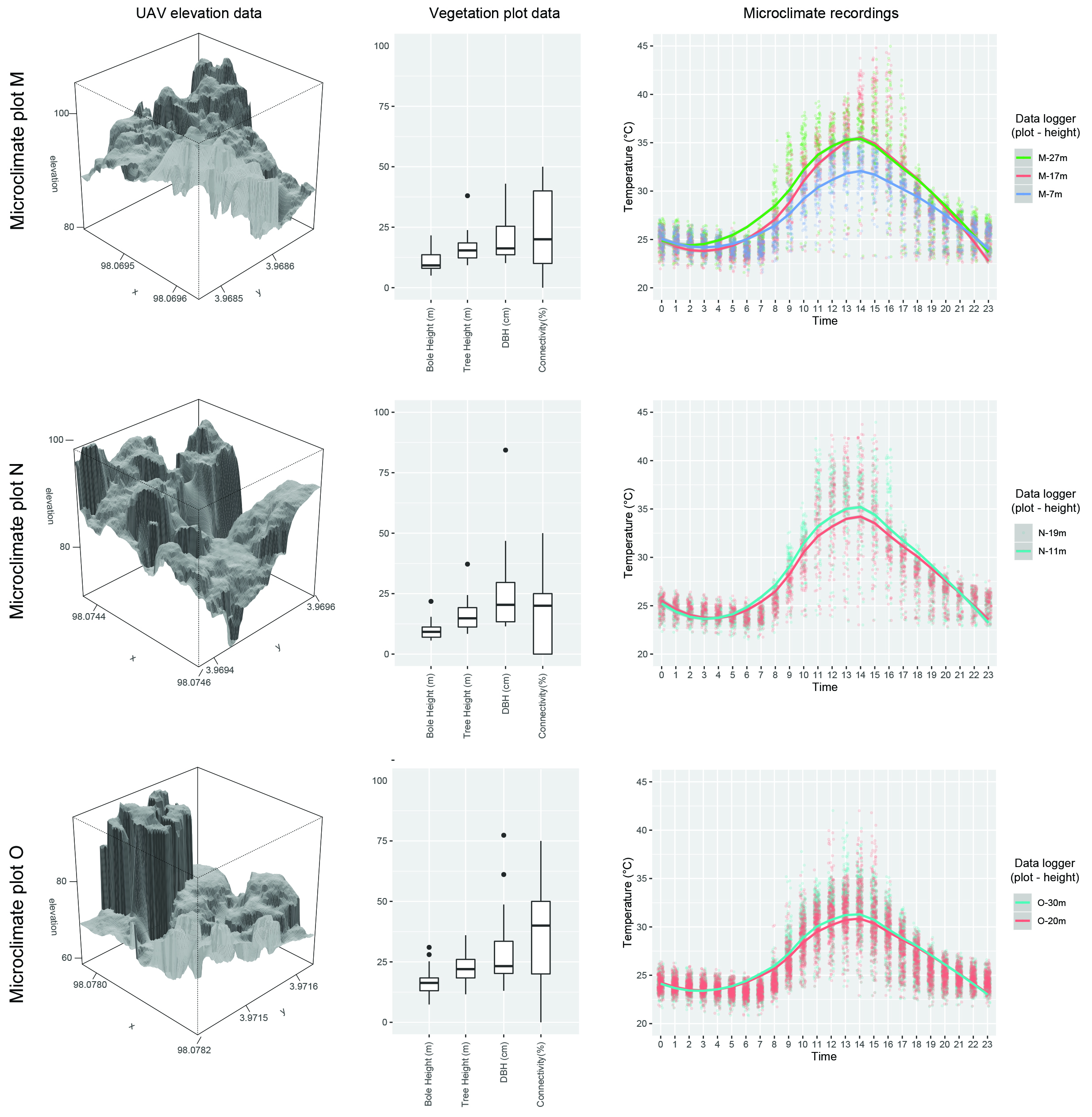


Figure 10d. Each plot’s UAV derived canopy topography, a sample of vegetation plot variables and temperatures recorded by dataloggers operating on the same dates


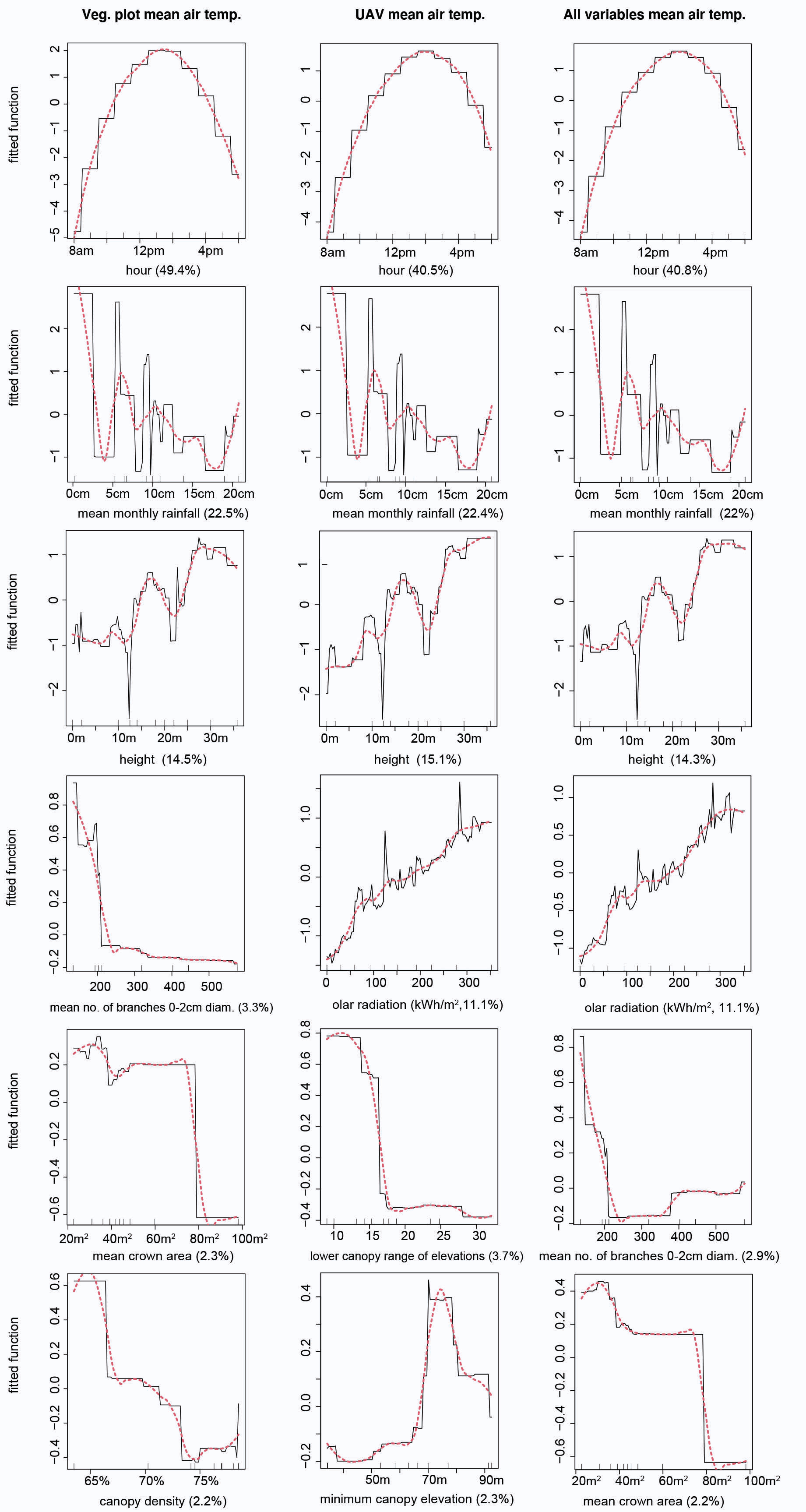


Figure 11a. Fitted functions (cantered by subtracting their mean value) of response variables of BRT models shown with smoothed trend lines. Note different scales along the y axis. Higher fitted function increase temperatures in models


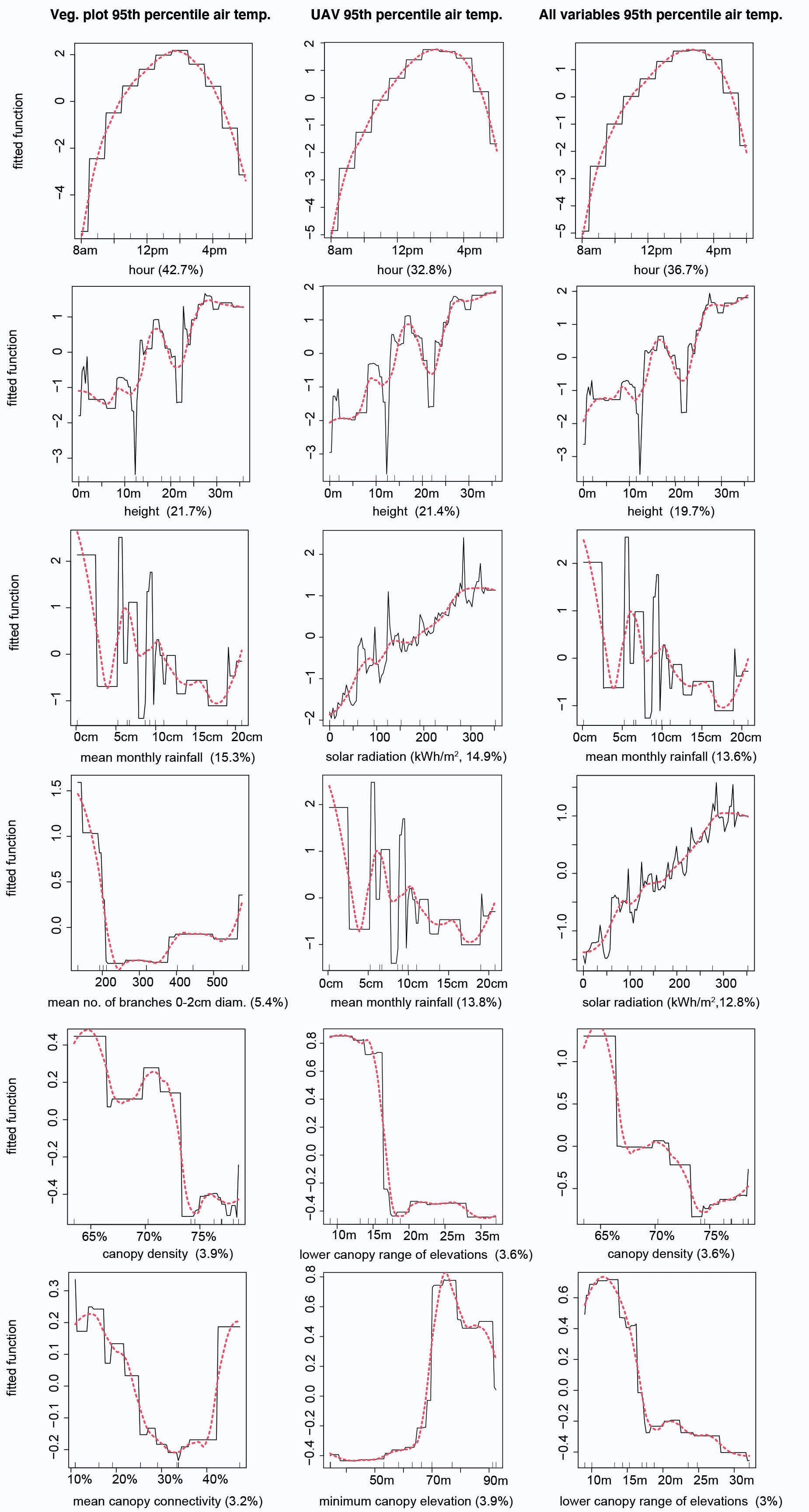


Figure 11b. Fitted functions (cantered by subtracting their mean value) of response variables of BRT models shown with smoothed trend lines. Note different scales along the y axis. Higher fitted function increase temperatures in models

| All variables mean air temp. | hour | data logger height | solar radiation | mean monthly rainfall | lower canopy range of elevations | relative canopy elevation | mean DBH | mean crown area | mean branches 0-2cm diam. | canopy density |
| --- | --- | --- | --- | --- | --- | --- | --- | --- | --- | --- |
| hour | 0 | 28.3 | 17.8 | 48.5 | 1.5 | 5.2 | 1.8 | 1.7 | 9.2 | 10.4 |
| data logger height | - | 0 | 116.8 | 17.8 | 6.9 | 6.0 | 70.9 | 2.4 | 7.3 | 4.4 |
| solar radiation | - | - | 0 | 40.9 | 2.9 | 33.1 | 4.2 | 4.1 | 4.7 | 65.8 |
| mean monthly rainfall | - | - | - | 0 | 2.0 | 10.6 | 0.9 | 3.6 | 3.7 | 11.9 |
| lower canopy range of elevations | - | - | - | - | 0 | 4.2 | 0.0 | 0.0 | 0.0 | 0.0 |
| relative canopy elevation | - | - | - | - | - | 0 | 1.2 | 0.0 | 0.1 | 0.1 |
| mean DBH | - | - | - | - | - | - | 0 | 0.0 | 0.0 | 0.0 |
| mean crown area | - | - | - | - | - | - | - | 0 | 0.0 | 0.2 |
| mean branches 0-2cm diam. | - | - | - | - | - | - | - | - | 0 | 0.1 |
| canopy density | - | - | - | - | - | - | - | - | - | 0 |

Table 1a. Interaction matrix of variables in BRT models, with the strongest interactions highlighted in green

| UAV mean air temp. | hour | data logger height | solar radiation | mean monthly rainfall | mean canopy elevation | stand. dev. of canopy elevations | minimum canopy elevation | lower canopy range of elevations | upper canopy range of elevations | relative canopy elevation |
| --- | --- | --- | --- | --- | --- | --- | --- | --- | --- | --- |
| hour | 0 | 27.0 | 7.4 | 58.3 | 0.3 | 1.6 | 1.3 | 1.9 | 0.1 | 5.2 |
| data logger height | - | 0 | 101.9 | 17.5 | 3.1 | 15.2 | 2.3 | 3.6 | 11.4 | 20.4 |
| solar radiation | - | - | 0 | 49.7 | 0.7 | 1.3 | 2.3 | 17.2 | 1.1 | 47.0 |
| mean monthly rainfall | - | - | - | 0 | 7.6 | 1.4 | 6.1 | 2.2 | 2.4 | 10.4 |
| mean canopy elevation | - | - | - | - | 0 | 0.2 | 0.0 | 0.3 | 0.0 | 3.2 |
| stand. dev. of canopy elevations | - | - | - | - | - | 0 | 0.0 | 0.0 | 0.1 | 0.4 |
| minimum canopy elevation | - | - | - | - | - | - | 0 | 0.8 | 0.0 | 1.3 |
| lower canopy range of elevations | - | - | - | - | - | - | - | 0 | 0.0 | 0.5 |
| upper canopy range of elevations | - | - | - | - | - | - | - | - | 0 | 0.0 |
| relative canopy elevation | - | - | - | - | - | - | - | - | - | 0 |

Table 1b. Interaction matrix of variables in BRT models, with the strongest interactions highlighted in green

| veg. plot mean air temp. | hour | data logger height | mean monthly rainfall | mean DBH | mean crown area | mean branches > 20cm diam. | mean branches 10-20cm diam. | mean branches 0-2cm diam. | mean crown connectivity | canopy density |
| --- | --- | --- | --- | --- | --- | --- | --- | --- | --- | --- |
| hour | 0 | 84.5 | 71.4 | 22.2 | 19.9 | 0.5 | 2.0 | 19.0 | 16.8 | 143.1 |
| data logger height | - | 0 | 14.2 | 60.4 | 3.0 | 1.7 | 14.3 | 5.5 | 11.5 | 1.5 |
| mean monthly rainfall | - | - | 0 | 1.4 | 2.3 | 1.2 | 1.2 | 3.1 | 2.1 | 13.3 |
| mean DBH | - | - | - | 0 | 0.0 | 0.0 | 0.0 | 0.0 | 0.0 | 0.0 |
| mean crown area | - | - | - | - | 0 | 0.1 | 0.2 | 0.0 | 0.0 | 0.0 |
| mean branches > 20cm diam. | - | - | - | - | - | 0 | 0.0 | 0.0 | 0.0 | 0.0 |
| mean branches 10-20cm diam. | - | - | - | - | - | - | 0 | 0.0 | 0.0 | 0.2 |
| mean branches 0-2cm diam. | - | - | - | - | - | - | - | 0 | 0.0 | 0.2 |
| mean crown connectivity | - | - | - | - | - | - | - | - | 0 | 0.0 |
| canopy density | - | - | - | - | - | - | - | - | - | 0 |

Table 1c. Interaction matrix of variables in BRT models, with the strongest interactions highlighted in green

| All variables 95th percentile air temp. | hour | data logger height | solar radiation | mean monthly rainfall | minimum canopy elevation | lower canopy range of elevations | relative canopy elevation | mean DBH | mean branches 0-2cm diam. | canopy density |
| --- | --- | --- | --- | --- | --- | --- | --- | --- | --- | --- |
| hour | 0 | 87.3 | 30.4 | 24.9 | 9.5 | 3.3 | 61.7 | 51.2 | 27.3 | 63.9 |
| data logger height | - | 0 | 325.8 | 19.7 | 6.0 | 8.7 | 17.7 | 162.3 | 12.7 | 20.4 |
| solar radiation | - | - | 0 | 23.8 | 4.1 | 11.7 | 72.5 | 26.9 | 2.8 | 218.6 |
| mean monthly rainfall | - | - | - | 0 | 2.4 | 1.4 | 29.2 | 3.4 | 0.8 | 49.9 |
| minimum canopy elevation | - | - | - | - | 0 | 0.0 | 2.5 | 0.2 | 0.0 | 0.0 |
| lower canopy range of elevations | - | - | - | - | - | 0 | 0.1 | 0.2 | 0.1 | 0.2 |
| relative canopy elevation | - | - | - | - | - | - | 0 | 0.2 | 0.1 | 0.1 |
| mean DBH | - | - | - | - | - | - | - | 0 | 0.1 | 0.0 |
| mean branches 0-2cm diam. | - | - | - | - | - | - | - | - | 0 | 0.1 |
| canopy density | - | - | - | - | - | - | - | - | - | 0 |

Table 1d. Interaction matrix of variables in BRT models, with the strongest interactions highlighted in green

| UAV 95th percentile air temp. | hour | data logger height | solar radiation | mean monthly rainfall | mean canopy elevation | stand. dev. of canopy elevations | minimum canopy elevation | lower canopy range of elevations | upper canopy range of elevations | relative canopy elevation |
| --- | --- | --- | --- | --- | --- | --- | --- | --- | --- | --- |
| hour | 0 | 96.0 | 26.6 | 25.7 | 1.9 | 6.2 | 6.5 | 12.2 | 1.1 | 32.6 |
| data logger height | - | 0 | 271.8 | 35.3 | 7.3 | 17.1 | 9.6 | 12.4 | 23.4 | 12.4 |
| solar radiation | - | - | 0 | 39.9 | 11.4 | 4.9 | 3.6 | 30.8 | 3.0 | 136.1 |
| mean monthly rainfall | - | - | - | 0 | 18.9 | 2.8 | 5.9 | 0.8 | 3.6 | 52.5 |
| mean canopy elevation | - | - | - | - | 0 | 0.8 | 0.0 | 0.1 | 0.1 | 6.3 |
| stand. dev. of canopy elevations | - | - | - | - | - | 0 | 0.2 | 0.0 | 0.1 | 1.6 |
| minimum canopy elevation | - | - | - | - | - | - | 0 | 0.1 | 0.0 | 1.9 |
| lower canopy range of elevations | - | - | - | - | - | - | - | 0 | 0.0 | 1.0 |
| upper canopy range of elevations | - | - | - | - | - | - | - | - | 0 | 0.5 |
| relative canopy elevation | - | - | - | - | - | - | - | - | - | 0 |

Table 1e. Interaction matrix of variables in BRT models, with the strongest interactions highlighted in green

| Veg. plot 95th percentile air temp. | hour | data logger height | mean monthly rainfall | mean DBH | mean tree height | mean branches 0-2cm diam. | mean crown connectivity | canopy density | cumulative crown area | tree height range |
| --- | --- | --- | --- | --- | --- | --- | --- | --- | --- | --- |
| hour | 0 | 143.6 | 31.1 | 80.9 | 15.4 | 31.2 | 54.7 | 414.8 | 5.1 | 18.0 |
| data logger height | - | 0 | 32.1 | 137.3 | 7.4 | 49.1 | 10.2 | 12.1 | 12.5 | 6.9 |
| mean monthly rainfall | - | - | 0 | 3.8 | 1.2 | 1.5 | 5.3 | 28.0 | 7.8 | 4.3 |
| mean DBH | - | - | - | 0 | 0.0 | 0.0 | 0.0 | 0.0 | 0.0 | 0.0 |
| mean tree height | - | - | - | - | 0 | 0.0 | 0.1 | 0.0 | 0.0 | 0.0 |
| mean branches 0-2cm diam. | - | - | - | - | - | 0 | 0.0 | 0.2 | 0.0 | 0.1 |
| mean crown connectivity | - | - | - | - | - | - | 0 | 0.0 | 0.0 | 0.0 |
| canopy density | - | - | - | - | - | - | - | 0 | 0.0 | 0.1 |
| cumulative crown area | - | - | - | - | - | - | - | - | 0 | 0.0 |
| tree height range | - | - | - | - | - | - | - | - | - | 0 |

Table 1f. Interaction matrix of variables in BRT models, with the strongest interactions highlighted in green





Figure 12. Projected mean microclimatic air temperature using a model with only UAV derived variables for the Sikundur area in July at 11am, 2pm and 5pm at a fixed height of 17m above ground
